# Standardized International Manual of the Fugl-Meyer Assessment of Motor Function After Stroke

**DOI:** 10.1177/15459683251412300

**Published:** 2026-03-03

**Authors:** Julie Hervé-Colas, Sarah P. Newton, Stefan T. Engelter, Kathryn S. Hayward, Jeremia P.O. Held, Nadine Intering, Gert Kwakkel, Johannes Pohl, Darcy S. Reisman, Anne Schwarz, Katharina S. Sunnerhagen, Janne Marieke Veerbeek, Karin Wiesner, Sarah B. Zandvliet, Margit Alt Murphy

**Affiliations:** 1 Departement of Physiotherapy, Geneva School of Health Sciences, University of Applied Sciences and Arts Western Switzerland, HES-SO, Geneva, Switzerland; 2 Melbourne School of Health Sciences, University of Melbourne, Melbourne, VIC, Australia; 3 Department of Occupational Therapy, Austin Health, Melbourne, VIC, Australia; 4 Department of Rehabilitation and Neurology, University Department of Geriatric Medicine FELIX PLATTER, University of Basel, Basel, Switzerland; 5 Departments of Physiotherapy and Medicine, University of Melbourne, Melbourne, VIC, Australia; 6 Clinical Trial Unit, Bellevue Medical Group, Zurich, Switzerland; 7 Department of Clinical Neurosciences, University Hospital Lausanne, Lausanne, Switzerland; 8 Faculty of Medicine and Biology, University of Lausanne, Lausanne, Switzerland; 9 Neuro X Institute, Ecole Polytechnique Féderale de Lausanne (EPFL), Lausanne, Switzerland; 10 Department of Rehabilitation Medicine, Amsterdam Movement Sciences, Amsterdam Neuroscience, Amsterdam University Medical Centers, Amsterdam, the Netherlands; 11 Department of Physical Therapy and Human Movement Sciences, Feinberg school of Medicine, Northwestern University, Chicago, IL, USA; 12 Lake Lucerne Institute, Data Analytics and Rehabilitation Technology (DART), Vitznau, Switzerland; 13 Department of Physical Therapy, University of Delaware, Newark, DE, USA; 14 Department of Neurology, David Geffen School of Medicine at UCLA, Los Angeles, CA, USA; 15 California Rehabilitation Institute, Los Angeles, CA, USA; 16 Department of Clinical Neuroscience, Institute of Neuroscience and Physiology, Sahlgrenska Academy, University of Gothenburg, Gothenburg, Sweden; 17 Clinic for Neurology and Neurorehabilitation, Luzerner Kantonsspital, University Teaching and Research Hospital of the University of Lucerne, Lucerne, Switzerland; 18 Department of Rehabilitation, Donders Institute for Brain, Cognition and Behavior, Radboud University Medical Center, Nijmegen, the Netherlands; 19 Erasmus MC, Department of Rehabilitation Medicine, University Medical Center Rotterdam, Rotterdam, the Netherlands; 20 Department of Clinical Neuroscience, Institute of Neuroscience and Physiology, University of Gothenburg, Gothenburg, Sweden; 21 Department of Health and Rehabilitation, Institute of Neuroscience and Physiology, Sahlgrenska Academy, University of Gothenburg, Gothenburg, Sweden

**Keywords:** Fugl-Meyer Assessment, motor assessment, international consensus manual, measurement properties, recommendations, outcome research

## Abstract

**Background:**

Fugl-Meyer Assessment of upper and lower extremity is the recommended primary clinical outcome measure of motor function in stroke rehabilitation. However, variations between different manuals undermine the reliability of the assessment, making it difficult to compare results and pool data across trials and centers.

**Objective:**

To develop a consensus-based international Fugl-Meyer Assessment (FMA) manual aligned with the original assessment along with agreed recommendations for assessor training. Additionally, the study aims to provide a critical review of current evidence on the measurement properties.

**Methods:**

Fourteen experienced health professionals and expert FMA users, working in 3 different continents, 6 countries and 13 research centers, participated in an iterative consensus process to generate a comprehensive agreed FMA manual. Agreement of at least 75% determined by voting was the minimum accepted threshold for each section of the manual. Consensus on assessor training was also sought. The evidence on measurement properties was compiled through a systematic literature review.

**Results:**

Greater than 79% agreement was reached for each section of the final FMA manual. Assessors should have strong foundation in neurorehabilitation and clinical assessment. Novice assessors should undergo structured training including supervised practical training with patients to ensure consistent and accurate scoring. Previous research adherent to the original FMA assessment, confirms strong validity and reliability, but provide limited information of minimal clinically important difference thresholds.

**Conclusions:**

This internationally agreed manual provides a common ground for improved consistency in administration of FMA worldwide and thereby will enable reliable data pooling and increase the comparability of results in future trials and clinical practice.

## Introduction

The Fugl-Meyer Assessment (FMA) was first introduced in 1975 by Fugl-Meyer et al^1^ and the original paper is 1 of the most cited in the field with approximately 4000 citations registered in the Web of Science database by 2025. The FMA is now the primary clinical outcome measure recommended for assessment of motor function in stroke rehabilitation clinical practice and research.^2-12^ The agreed recommendations to include the FMA as an outcome measure in each stroke trial, have facilitated a more standardized approach to measurement in stroke recovery and rehabilitation research and clinical practice, paving the way for more homogeneous characterization of patient cohorts, stroke recovery patterns and trial outcomes. To support the uptake of these recommendations, the suggested outcome measures should be implemented in a standardized manner across research and clinical centers. Agreed protocols and manuals are a prerequisite to enable comparison between studies, facilitate meta-analyses and data aggregation and thereby strengthen the evidence-base for stroke rehabilitation interventions.^7,13,14^

The conceptual framework underlying the FMA motor assessment is based on the observed stages of recovery in voluntary motor function.^1^ These stages reflect stereotypical synergistic muscle activation patterns of the upper and lower limb, originally described by Twitchell and Brunnstrom.^1,15,16^ The synergy dependence is assessed by the patient’s ability to move a single joint without simultaneously moving other joints (Box 1). The concept of synergy dependence is applied to movements of the shoulder and elbow alone, as recovery of wrist and hand function can occur independent of arm recovery.^1,17,18^ More recent research have demonstrated that apart from reflex activity items, the FMA motor assessment represents the unidimensional construct of the motor function.^19,20^ The standardized description of body positions during movement execution while restricting compensatory movements assures that the FMA is measuring body functions as defined by the International Classification of Functioning, Disability and Health (ICF).^21^

The FMA for the upper (FMA-UE) and lower extremity (FMA-LE) were originally developed concurrently in both English and Swedish,^1,22^ with official translations now available in multiple languages.^23-27^ In addition to motor function assessment, the original FMA included assessment of sensation, pain and passive range of motion, also referred to as non-motor scores.^1^ However, the non-motor sections are commonly used to characterize patients rather than evaluative outcomes.^1,28^ The original FMA also included a section assessing balance, but due to weaker measurement properties, it has not been widely adopted.^28,29^

The measurement properties including validity and reliability of the FMA have been tested and reported as excellent in multiple studies.^30-33^ However, the early reliability evaluations were predominantly performed in small samples (<30 participants) using parametric statistics (eg, intraclass correlation coefficients).^29,34-36^ In more recent studies with larger sample sizes,^30,31,37,38^ the use of parametric analyses still dominates, although there are a handful of studies that evaluate reliability using statistics more suited for an ordinal scale (eg, weighted Kappa and other rank-based analyses).^26,32,33,39,40^ The validity of FMA has additionally been confirmed using techniques such as Rasch analysis, mathematical modeling for ordinal level of measurements, which has proven unidimensional item-level measurement properties for all motor items.^20,41^ While it is acknowledged that the reflex items of the FMA are measuring a different construct, they do not seem to influence the motor impairment presentation.^42^

**Central concepts and definitions related to FMA**

**Muscle synergies** after stroke can be defined as pathological stereotypical fixed patterns of co-activation across a set of muscles during a voluntary movement attempt of the upper or lower extremity;^1,47,48^ typically described as flexion or extension synergy presented in the upper extremity as simultaneous shoulder abduction, elbow flexion, and forearm supination or shoulder adduction, elbow extension, and forearm pronation, respectively; and in the lower extremity as simultaneous hip flexion, knee flexion, and foot dorsal flexion or hip extension, knee extension, and foot plantar flexion, respectively.^1^

**Synergy dependence** indicates a loss of independent joint movement control during a voluntary movement attempt and evolves from initial dependence (movements within synergies) or partial dependence (movement with mixed synergies) to complete voluntary movement with normal muscle activation patterns (movement with little or no synergy dependence).^1^

**Muscle co-activation** after stroke is typically seen as a pathological co-activation of agonist and antagonist muscles and/or co-activation of supplementary agonists muscles to compensate for muscle weakness.^47,49^

**Motor function and impairment** are the positive and negative terms of body functions according to the International Classification of Functioning, Disability and Health (ICF) terminology, defined as physiological functions of body systems.^21^

Trial or site-specific manuals with training and credentialing of assessors are commonly used to reach a high level of intra- and interrater reliability.^29,30,36^ These procedures are time-consuming and require extra resources,^43,44^ and when conducted individually, discrepancies between multiple manuals can occur.^45,46^ Access to an agreed international manual will significantly decrease the costs and time spent on producing manuals for individual trials, and more importantly ensure a more consistent application.

This initiative brought together international experts with extensive knowledge and experience in FMA use in stroke research or clinical practice. The primary aim was to provide a consensus-based comprehensive international manual of the FMA-UE and FMA-LE motor assessment that adheres closely to the original assessment along with an agreed recommendation for structured assessor training applicable worldwide. The secondary aim was to provide a critical review of current evidence on measurement properties and motor impairment severity categories of the FMA with a focus on studies following the original FMA assessment.

## Methods

### FMA Manual Development—Part 1

Experts from established international research centers with experience using FMA in clinical trials or observational studies were invited to the group. The initial members were identified by the 2 authors (M.A.M., J.H.C.). Subsequently a purposeful snowball method was used to identify experts through the respective professional international networks of the included experts. The aim was to compose an expert group of 10 to 15 members with a representation from different health professionals and research sites. The 14 experts who agreed to participate had all been or were involved in the development of standardized FMA manuals for specific clinical trials, studies or clinical centers at their respective sites. Ten experts were primarly affiliated with sites in Europe (Sweden, Netherlands, Switzerland), 2 in Australasia (Australia) and 2 in North America (USA). The expert group included 11 physiotherapists, 1 occupational therapist and 2 medical doctors who specialized in rehabilitation medicine or neurology. All had substantial experience in stroke rehabilitation and the application of the FMA in clinical practice and research, with experience ranging from 4 to 25 years (median 10 years). All but 1 of the experts were experienced with the actual administration of the FMA. One additional senior physiotherapist (NI) with theoretical but no clinical experience in application of FMA, provided insights from a novel user perspective during the manual development process. Two senior physiotherapists, with expertise in administering and scoring the originally developed FMA^1,22^ were additionally consulted prior to discussion of critical points.

The FMA manual consensus process was initiated in April 2024 by the 2 coordinating authors (M.A.M., J.H.C.) and included 3 iterative rounds of revisions conducted via email. Two online meetings were organized for the whole group to agree on the main steps and procedures, as well as to discuss critical points of the work. The first draft was based on the information available in the original paper by Fugl-Meyer et al^1^ complemented by descriptions in the Swedish published manual.^22^ For the remaining ambiguities, previously published detailed manuals were screened to gather additional information and support discussion of specific critical points (Supplemental Tables 1 and 2).^29,30,36,50-52^

The first FMA manual draft and all consecutive revisions were conducted by the coordinating authors. Individual feedback, comments and suggestions provided by the expert group members were collated and circulated to the whole group along with comments and suggestions for revised versions at each round. This process was repeated until all points had been discussed (on meetings or by e-mails) thoroughly and no new comments were received. The final version was then deemed to be ready for independent voting through e-mail. For each section, votes (agreed or not), comments and suggestions for changes were collated. Agreement of at least 75% was set as the minimum accepted threshold for each section, similar to formal Delphi processes.^53^ The votes, along with suggested revisions, were only sent back to the 2 coordinating authors who summarized the results and suggestions back to the whole group anonymously. The voting was first conducted on the FMA-UE followed by the FMA-LE. A final language and layout proofreading of the manual was done by 2 native English speakers (S.P.N., K.S.H.).

Additionally, a separate iterative review process among the experts was used to develop a consensus-based recommendation on FMA assessor training. These recommendations were primarily targeted for trial researchers but can also be used in clinical settings. Suggestions, collected from all experts, were iteratively circulated by e-mail for feedback and revisions until a consensus was reached on the final statements included in the recommendation statement.

### Evidence of Measurement Properties and Severity Categories—Part 2

A systematic search was done in PubMed using the search terms (Fugl-Meyer Assessment) AND (stroke) AND (validity OR reliability OR responsiveness). The search was limited to title and abstract and included truncated versions of the search terms. Eligible studies needed to include at least 30 participants with stroke and report results on FMA construct validity, reliability or responsiveness. The screening process was performed independently by 2 researchers (M.A.M., J.H.C.) and disagreements were discussed with a third reviewer (S.P.N.). To identify other potentially relevant studies the reference lists of the included studies were screened. From the included studies, stroke population characteristics, floor and ceiling effects, reliability, validity and responsiveness indicators (minimal detectable difference, MDC and minimal clinically important difference, MCID) were extracted and presented descriptively. The FMA instruction manual applied in the study was documented. In addition, relevant studies defining impairment severity levels, such as severe, moderate and mild, by the FMA-UE or FMA-LE scores retrieved from the literature search or personal libraries were identified and summarized.

## Results

### Agreed FMA Manual—Part 1

The results of the expert group consensus voting process are shown in Table 1. The voting on the FMA-UE and FMA-LE manuals reached a median agreement of 89% (range 79%-100%) and 93% (range 79%-100%), respectively. All expert group members also agreed on the final proposed recommendations for FMA assessor training.

**Table 1.** Percentage of Agreement Across 14 Experts for Each Section of the Fugl-Meyer Assessment Manual.

Fugl-Meyer Assessment Manual sections	Percentage of agreement
Fugl-Meyer assessment of upper extremity	
General instructions	79
A. I. Reflex activity	100
A. II. Movements within synergies	93
A. III. Movements with mixed synergies	100
A. IV. Movements with little or no synergy dependence	93
A. V. Normal reflex activity	100
B. Wrist	79
C. Hand	79
D. Coordination—speed	86
Quick guide on assistance and support	86
Fugl-Meyer assessment of lower extremity	
Assessment sheet	100
General instructions	100
E. I. Reflex activity	100
E. II. Movements within synergies	93
E. III. Movements with mixed synergies	79
E. IV. Movements with little or no synergy dependence	86
E. V. Normal reflex activity	100
F. Coordination—speed	93
Quick guide on assistance and support	86

During the iterative rounds, several critical points were identified in which the administration of specific items varied among experts (Supplemental Table 3). These variations could largely be attributed to previous training and FMA experience as well as insufficient or unclear item descriptions in the original paper.^1^ For the FMA-UE, discrepancies in administration were found for start positions of shoulder flexion (Item 17), how strictly the full elbow extension was interpreted in scoring (Item 13, 15-17, 21, 22), the hand position required for cylinder and spherical grasp items (Item 29 and 30) and in wrist circumduction (Item 23). The expert group agreed that an extension deficit of up to 30° due to elbow joint contraction can be considered as the patient’s maximum available joint range to allow assessment in items that call for a full elbow extension as the starting position. Likewise, it was unanimously agreed that a full passive 180° of shoulder flexion is hardly attainable in elderly stroke patients, and instead a population-based reference of 160° of passive joint range^54^ can be considered a full range. For the wrist circumduction, cylinder and spherical grip items, the original manuals^1,22^ along with experienced senior users familiar with the original assessment, were consulted to reach a decision (Supplemental Table 3).

The FMA-LE administration discrepancies between experts were fewer. Some clarifications were made during the iterative revision to improve consistency in administration. It was clarified that assessment of passive range of motion is performed in the same positions as the item is tested, the scoring of the ankle movements should preferably be performed without shoes and that the assessor needs to ensure that knee flexion beyond 90° is active.

A section outlining general instructions was added to provide explicit guidance regarding test order, number of trial attempts and how to manage deviations from the correct standard testing position, for example, sitting in acute hospital bed rather than chair. The experts agreed to allow deviations from the correct testing position if the assessor can ensure that accurate scoring can still be made. In addition, the maximum allowed number of attempts at an item was suggested to be kept low (1-3 attempts). The instructions on the allowed assistance and support during the assessment followed guidance provided in the original manuals. Note that no physical assistance is provided by the assessor to the specific movement that is assessed.

The agreed FMA-UE and FMA-LE of motor function is available for free use for researchers and clinicians (Supplemental material 4 and 5). Instructional videos in accordance with the developed manual are available at University of Gothenburg homepage https://www.gu.se/en/neuroscience-physiology/fugl-meyer-assessment.

### Recommendations for Assessor Training

To ensure validity and reliability in FMA scoring, assessors should have a strong foundation in neurorehabilitation and clinical assessment (ie, sufficient training and experience in patient assessment). The most suitable professionals for this role include physiotherapists and occupational therapists with expertise in neurorehabilitation as well as neurologists or stroke physicians with rehabilitation training.

Novice FMA assessors (otherwise trained in neurorehabilitation) should undergo structured training, including both didactic and practical components of administration and scoring, by an experienced assessor covering the basic principles of assessment and scoring. The practical training should include video-based examples and/or training sessions with patients at various recovery stages and severity levels (severe, moderate and mild). After the initial structured training, each assessor is advised to conduct at least 3 independent assessments in patients with stroke. Supervision by an experienced assessor needs to be available either during or after the assessments to facilitate discussion of potential critical points and challenges. For continuous competency, peer-training is recommended on a regular basis (eg, annually) to maintain scoring consistency.

In clinical trials, the content of assessor training should be defined and operationalized in the trial protocols. Within each trial there should be an assigned expert therapist(s) to oversee and coordinate assessor training across participating sites.

### Current Evidence on Measurement Properties of the FMA—Part 2

**Figure 1.**
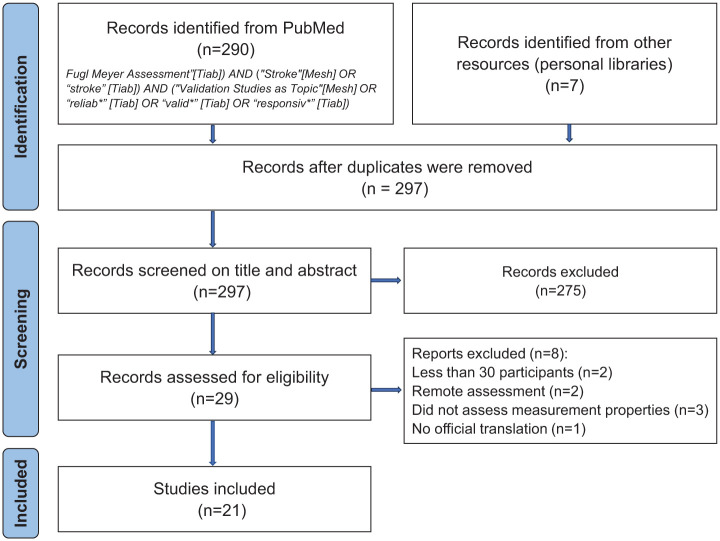
PRISMA flowchart of the literature search.

The results from the systematic literature search yielded 290 records, from which 21 studies were included for data extraction (Figure 1). Seventeen studies reported data on FMA-UE and 10 studies on FMA-LE. The majority of the studies followed the original FMA manual,^1^ while 3 FMA-UE^30,31,55^ and 1 FMA-LE^56^ studies applied other published manuals. The extracted data on measurement properties are summarized in Tables 2 and 3.

**Table 2.** Measurement Properties of FMA-UE in Studies With More Than 30 Participants.

Study	Sample (n) and stage of stroke	Time post stroke, mean (SD)	FMA score, mean (SD)	Measurement properties
Rabadi 2006^57^ USA	100 subacute	16 (9) d	30 (24)	Correlation with ARAT .77-.88
Hsueh 2008^37^ Taiwan	55 subacute 60 chronic	18.6 (12) d; ≥1 y	NR	Floor 0-4%; Ceiling 2-9% (subacute) Intrarater: ICC .98 (.96); SRD 7.2p (chronic)
Hsieh 2009^58^ Taiwan	57 chronic	13 (7.6) mo	49 (9)	Correlation with ARAT .73, WMFT .71-.76
Lin 2009^38^ Taiwan	30 subacute 30 chronic (reliability)	14, 90, 180 d 12 (2) mo	32 (26)	Floor 9%, 2%; Ceiling: 6%, 17% (subacute, chronic) Intrarater: ICC = .99 (.99), MDC 5p (chronic) Interrater: ICC = .96 (.92), MDC 13p (subacute) Correlation with ARAT .90-.92, WMFT .93-.94, STREAM .94-.96
Arya 2011^59^ India	71 early subacute 4w task-specific arm training	8.4 (5.3) wk	14 (11)	MCID 9p (AUC .84) mRS (≥1 p change) MCID 10p (AUC .98) GRPC (meaningful improvement)
Kim 2012^60^ Korea	50 chronic (video)	2.4 (1.8) y	60 (25)	Intrarater: ICC = 0.97 (.94); SRD 9p Interrater: ICC = 1.0 (1.0) Correlation with Jebson Taylor .76, grip strength .72, MAS .69
Page 2012^61^ USA	146 chronic 6w task-specific arm training	66 (63) mo	37-40 (6)	MCID 4-7p (AUC .61-.70) therapist rating on 5 arm activities MCID 5p (AUC .65) therapist rating of overall arm function (excellent improvement >50% on GRPC)
See 2013^30^ USA^a^	35 chronic	54 (47) mo	31 (15)	Intarater: ICC .99 (−), MDC_90_ 3.2p Correlations with ARAT .93, BBT .86, 9HPT .75, grip strength .88, SIS Hand .86
Lundquist 2016^31^ Denmark^a^	50 subacute 3w inpatient rehabilitation	13.7 (9) d	30 (21)	Floor 0; Ceiling 0 Intrarater: ICC .95 (.93) Interrater ICC .95 (.93) Correlation with MAS-UE .94 MCID 4p (AUC .87) GRPC (any perceived improvement)
Amano 2018^55^ Japan^a^	30 chronic	41 (1, 79) mo median (Q1, Q3)	44 (23, 59) median (Q1, Q3)	Floor 0, Ceiling 10% Intrarater wKappa 1.0 (1.0), wKappa 1.0 (1.0); PA 100% (video) Interrater ICC .98 (.93); wKappa .84 (.67), PA 87%; Subscale wKappa .82-.86 (.65-.73) Correlation with ARAT .95, BBT .95, MAL .93
Hernandez 2019^40^ Colombia	60 subacute	12 (10) d	58 (48, 65) median (Q1, Q3)	Floor 0; Ceiling 22% Intrarater: Item PA 79%-100%; Total score ±3-points PA 78%-82% Interrater: Item PA 90%-100%: Total score ±1-point PA 80%-83%
Ikram 2021^26^ Pakistan	50 chronic	6-12 mo	NR	Intrarater: Item wKappa .85-.97 Interrater: Item wKappa .84-.95 Correlation with mRS .69, NIHSS .79, FIM .70
Kim 2021^62^ Korea	31 chronic	11.2 (7.9) mo	24 (12)	Intrarater: ICC .96-.98 (−) Interrater: ICC .98 (−) Correlation with MI .78, MAS .56,
Hochleitner 2023^32^ Italy	60 subacute	18 (9.8) d	55 (36, 61) median (Q1, Q3)	Floor 0; Ceiling 6.7% Intrarater: Item PA 72%-100%; Total ±2-points PA 73%-86% Interrater: Item PA 72%-100%; Total ±2-points PA 88%-92%
Huynh 2023^63^ USA	51 early subacute 6w usual care	4.7 (2.3) d	12 (13)	Intrarater: MDC 7p MCID 13p (AUC .83) (perceived decrease in arm weakness of 3-points on a scale 0-10); MCID 9p (AUC .70) mRS (≥1 p change)
Wiesner 2024^33^ Switzerland	50 subacute	90 (14) d	40 (28, 51) median (Q1, Q3)	Interrater: ICC .98 (.96); Item PA 44-98%; Item wKappa .51-.98 (.27); Item Gwet .58-.98 (.44); Subscale wKappa .88-.91 (.84)
Pohl 2025^64^ Switzerland	60–93 early subacute	3 (2,4) d median (Q1,Q3)	22 (7, 37) median (Q1, Q3)	Floor 17% (day 3); Ceiling 0 MCID 7p (day 3-10); 4p (day 10-28); 7p (day 28-90) (AUC .68-.88) GRPC (meaningful improvement)

Abbreviations: ARAT, action research arm test; AUC, area under curve; BI, Barthel Index; BBT, box and block test; FIM, functional independence measure; FMA-UE, Fugl-Meyer upper extremity; GRPC, global rating of perceived change; ICC, intraclass correlation coefficient; MAL, motor activity log; MAS-UE, Motor Assessment Scale Upper Extremity; MCID, minimal clinically important difference; MDC, minimal detectable change; MI, Motricity Index; mRS, modified Ranking Scale; NIHSS, National Institutes of Health Stroke Scale; NR, not reported; PA, percentage of agreement; SRM, standardized response mean; SIS, Stroke Impact Scale; SRD, smallest real difference; STREAM, Stroke Rehabilitation Assessment of Movement; WMFT, Wolf Motor Function Test; w, week; wKappa, weighted kappa; 9HPT, 9-hole peg test.

a A modified version of the FMA-UE was used. For ICC, MDC, weighted Kappa and Gwet the lower confidence interval is shown in brackets when it was reported in the study.

**Table 3.** Measurement Properties of FMA-LE in Studies With More Than 30 Participants.

Study	Sample (n)	Time post stroke, mean (SD)	FMA, mean (SD)	Measurement properties
Beckerman 1996^65^ Netherlands	49 chronic	≥6 mo	18 (5)	Intrarater: ICC .86; SRD 5p
Hsueh 2008^37^ Taiwan	60 chronic 55 subacute	18.6 (12) days; ≥1 y	NR	Intrarater: ICC .95 (.91), SRD 3.8p
Hiengkaew 2012^66^ Thailand	61 chronic	40 (34) mo	22 (5)	Intrarater: ICC .94 (.89), MDC 3.6p
Kim 2012^60^ Korea	50 chronic (video)	2.4 (1.8) y	21 (8)	Intrarater: ICC = 0.87 (.76); SRD 8p Interrater: ICC = 0.93 (.89); SRD 6p Correlation with BBS .66
Pandian 2016^56^ India^a^	65 chronic 30 sessions (3x/week) leg/gait training	16 (6) mo	20 (4)	MCID 6p (AUC .98) FAC (≥1-point change) MCID 6p (AUC .93) GRS (meaningful improvement)
Hernandez 2019^39^ Colombia	60 subacute	12 (10) d	29 (26, 31) median (Q1, Q3)	Floor: 0; Ceiling: 15% Intrarater: Item PA 75%-100%; Total ±2-point PA 82%-92% Interrater: Item PA 88%-100% Total PA 75%-80%
Ikram 2021^26^ Pakistan	50 chronic	6-12 mo	NR	Intrarater: Item wKappa .75-.95 Interrater: wKappa .76-.95 Correlation with mRS .69, NIHSS .79, FIM .70
Kim 2021^62^ Korea	31 chronic	11.2 (7.9) mo	20 (6)	Intrarater: ICC .80-.91 Interater: ICC .88-.95 Correlation with MI .86, MAS .55, BBS .32
Hochleitner 2023^32^ Switzerland	60 subacute	18 (9.8) d	27 (23, 30) median (Q1, Q3)	Floor 0; Ceiling 6.7% Intrarater: Item PA 82%-100% Total ±1-points PA 73%-82% Interrater: Item PA 92%-100% Total 78%-82%
Wiesner 2024^33^ Switzerland	50 subacute	90 (14) d	24 (21, 28) median (Q1, Q3)	Interrater: ICC .85 (.70), Item PA 44%-100%, Item wKappa 0.30-0.74 (.04), Item Gwet .42-.97 (.19); Subscale (coordination) wKappa .62 (.42)

Abbreviations: AUC, area under curve; BI, Barthel Index; BBS, Bergs Balance Scale; FAC, Functional Ambulatory Categories; FIM, Functional Independence Measure; FMA-LE, Fugl-Meyer Lower Extremity; GRC, Global Rating Scale; ICC, intraclass correlation coefficient; MAS, Motor Assessment Scale; MCID, minimal clinically important difference; MDC, minimal detectable change (95%); MI, Motricity Index; mRS, modified Ranking Scale; NIHSS, National Institutes of Health Stroke Scale; NR, not reported; PA, percentage of agreement; SRM, standardized response mean; SRD, smallest real difference; w, week; wKappa, weighted kappa.

a A modified version of the FMA-LE was used. For ICC, MDC, weighted Kappa and Gwet the lower confidence interval is shown in brackets when it was reported in the study.

Most of the studies using the original FMA assessment,^1^ reported floor and ceiling effects below or equal with 15% both in the subacute and chronic stage of stroke, while 3 studies reported values between 17% and 22%.^38,40,57^ The original FMA construct validity is well established for multiple upper and lower extremity motor function and activity capacity outcomes. Excellent reliability was reported both for the FMA-UE (ICC ≥ 0.95, weighted Kappa ≥ 0.84) and FMA-LE (ICC ≥ 0.8, weighted Kappa ≥ 0.75) in studies adherent to the original assessment (Tables 2 and 3). The intra- and inter-rater agreement was reported as sufficient at the item level (percentage of agreement ≥ 72%, weighted Kappa ≥ 0.75), while a 1 to 3-point difference between the raters was needed to reach 70% agreement for the total score.^32,39,40^ The item level inter-rater agreement reached a median of 77% (range 44%-100%, weighted Kappa of 0.69) in subacute stroke in video-based ratings.^33^ For studies following the original FMA assessment, the FMA-UE MCID ranged from 4 to 13 points in the early subacute phase^59,63,64^ and from 4 to 7 points in the chronic phase.^61^ For the FMA-LE, an MCID of 6 points was reported for chronic stroke alone,^56^ although this study followed another published FMA protocol.

### Severity of Upper Extremity Impairment

The severity of upper extremity impairment is commonly classified as severe, moderate or mild in stroke trials, although specific cut-offs between studies vary (Table 4). Two studies using Receiver Operating Characteristic analysis provided FMA-UE cut-off scores associated with the activity capacity categories of the Action Research Arm Test (ARAT).^67,68^ One study, using hierarchical cluster analysis, reported an initial 3-group classification for the FMA-UE, which was refined to a 4-group classification to minimize overlap.^42^ A clinical anchor, defined as the capacity to complete a drinking task with the affected arm, was used in the SALGOT-study to discriminate between severe and moderate arm impairment,^69-72^ whereas mild impairment was defined as the ability to use the affected arm routinely in activities of daily living.^73^ No study was identified that reported the FMA-LE severity categories.

**Table 4.** Reported Cut-offs of Severity for FMA-UE Motor Function.

Published studies	Cut-offs for motor function/impairment severity				
Hoonhorst 2015^67^	No	Poor	Limited	Notable	Full
Chronic stage	0-22	23-31	32-47	48-52	53-66
Valladares 2024^68^	No	Poor	Limited	Notable	Full
Acute/early subacute	0-19	20-32	33-47	48-57	58-66
Subacute	0-19	20-30	31-47	48-55	56-66
Chronic	0-19	20-28	29-45	46-53	54-66
Woytowicz 2017^42^	Severe		Moderate		Mild
Chronic stroke	0-28		29-42		43-66
Woytowicz 2017^42^	Severe	Severe-moderate		Moderate-Mild	Mild
Chronic stroke	0-15	16-34		35-53	54-66
SALGOT-study^69,72^	Severe		Moderate		Mild
Acute to chronic	0-31		32-57		58-66
Pang 2006^63^	Severe		Moderate		Mild
Chronic	0-27		28-57		58-66

Acute/early subacute = median 9 days (range 6-30); Subacute = median 93 days (range 76-163); Acute to chronic = range from 3 days to 12 months.

## Discussion

A comprehensive consensus approach was undertaken by an international group of experts in stroke rehabilitation to compile an agreed FMA manual of motor function. The aim of this consensus work was to improve the consistency in administration across different sites and professionals, while remaining as close as possible to the original description of the assessment. The agreed manual provides detailed instructions, complemented with photos, on how to administer and score the items of the assessment. A median expert agreement of 89% and 93% was reached for the FMA-UE and FMA-LE manuals, respectively. In addition, a consensus recommendation for assessor training was reached among the experts. The evidence on validity and reliability estimates of the original FMA are robust, based on the studies following the original assessment instructions, while information on MCID thresholds is limited.

The current work demonstrates that despite discrepancies in the interpretation of the original FMA instructions, consensus was reached among experts working at different sites, regions, and continents. The transparent iterative process with multiple rounds of discussions and revisions helped to identify the critical points of discrepancy so they could be addressed. Returning to the original descriptions, and consulting experienced senior users familiar with the original assessment instructions, were crucial in solving discrepancies and staying true to the original administration. It was important to balance the level of detail provided in the manual to avoid irrelevant instructions (eg, measurements with a goniometer) while still specifying all critical points identified during discussions. In practice, assessors are likely to observe patients completing various movement strategies making the assessment challenging even with a comprehensive manual in hand. The recommendation for assessors in these situations is to rely on the core principle of the FMA: to assess active voluntary movement without compensation by other body parts.

To ensure correct and reliable assessment, sufficient experience in motor function assessment is required. Currently, there is limited research evidence available to provide guidance on the required training for conducting the FMA. Thus, the proposed recommendation for FMA assessor training is based on the clinical and research experience of the authors. It is well recognized that in clinical trials the assessors might not always have specific training in FMA assessment. In these cases, additional training is needed to ensure correct administration and scoring. For example, experience in conducting other assessments, such as the modified Ranking Scale or National Institutes of Health Stroke Scale, would not be considered sufficient experience. Likewise, we do not recommend that professionals without basic training in neurorehabilitation are recruited as FMA assessors in clinical trials.

For the novice FMA assessors, we suggest structured training, including practical sessions with either video-based or in-person patient assessments. A previous study showed that standardized training with patient videos lead to improved scoring accuracy among physiotherapy students (novice assessors).^30^ The improved accuracy reduced the variance in FMA-UE scores by 20%, which was pointed out as a potential source of increased statistical power.^30^ In the LEAPS (Locomotor Experience Applied Post Stroke) trial, assessor training included on-site coaching, feedback, practice for 1 month and annual monitoring for the duration of the trial. To serve as a trial assessor, a 90% accuracy compared to an expert’s score was required.^29^ High intra- and interrater reliability was reported for the trial assessors, although the 95% CI of the difference from the expert score was close to 6 points for FMA-UE and FMA-LE each.^29^ Assessor training was also evaluated in the AVERT-DOSE trial, including 258 assessors from 7 countries.^44^ The training materials included a manual, an instructional video and 2 patient videos to be scored. The results showed that many of the assessors (66%-74%) required additional training to achieve 90% scoring accuracy.^44^ Collectively, these evaluations of assessor training confirm that specific standardized assessor training is a necessity for any trial to achieve high-level consistency and accuracy between assessors. In line with previous work,^29,43^ we also recommend having procedures in place for continuous competency training in research trials that reach over several months and years.

The systematic literature review showed that the evidence base on validity and reliability is strong for studies following the original FMA instruction. Only 4 out of 21 studies referred to other trial-based protocols.^30,31,55,56^ The developed comprehensive manuals of the FMA-UE and FMA-LE adhere closely to the original assessment, which implies that the validity and reliability estimates will likely be in the same range as reported in these previous studies. Several studies also used the same instruction videos for assessor training as are recommended in the current work.^32,33,39,40^ In 3 of these studies, the reliability was evaluated between assessors working in the same center,^32,39,40^ and in another, 2 independent assessors from each trial site conducted the assessments.^33^ In future studies, the inter-rater reliability across multiple raters and sites should be evaluated to have a rough estimate for consistency and agreement applicable for international multicenter trials.

In evaluation of agreement between 2 or more assessors or when a comparison is performed with a correct score, data from previous studies can be used to set a minimum threshold required to ensure acceptable consistency. In the LEAPS trial, the discrepancy from the expert’s correct score was found to be approximately 6 points, both for the FMA-UE and FMA-LE. In previous reliability studies, an agreement above 80% was reached between the assessors when a 3-point difference was accepted in the FMA-UE and FMA-LE.^32,39,40^ These studies suggest that a maximum of 3-points discrepancy between the assessors can preferably be used as an estimate for future trials to reach at least 80% agreement between the assessors. This estimate will then be below the currently estimated MCID thresholds as well as the estimated minimal detectable change (Tables 2 and 3).

We also collated data on MCID as reported in studies using the original FMA instructions. For the FMA-UE, 3 studies reported MCIDs for the early subacute stage post stroke.^59,63,64^ Two of these studies reported MCIDs anchored to patient perceived meaningful improvement.^59,64^ One study with mild-to-moderate impairment reported that an improvement between 4 and 7 points early subacute was indicative of a meaningful change,^64^ while another study in severe motor impairment reported that a 10-point improvement early subacute was associated with meaningful change.^59^ In a study, in chronic stroke, a therapist rated improvement (≥50% improvement) was associated with a 4 to 7-point change in FMA-UE in individuals with moderate-to-mild motor impairment.^61^ The MCID of 9 points was indicative of the therapist-reported change (1-point change on the modified Ranking Scale) in the early subacute stage of stroke.^59,63^ These results suggest that a meaningful change in FMA-UE can vary from 4 to 10 points (6%-15% of the maximum 66 points) depending on the specific characteristics of the patient, stage of stroke and what criteria are used as an anchor. For the FMA-LE, the MCID value of 6 points (18% of the maximum 34 points) was reported in a single study in the chronic stage of stroke.

### Strengths and Limitations

This work did not aim to make changes to the original FMA but rather find consensus among different interpretations of the original scale. The iterative and transparent methodology used to develop this comprehensive FMA manual, strengthened the consensus process. The expert group had extensive experience in the field of stroke rehabilitation, including conducting and/or participating in national and international stroke trials employing FMA as an outcome measure. Experts with clinical training in physiotherapy, occupational therapy and rehabilitation medicine from 3 continents, 6 countries and 13 research centers were represented in the group. While this diversity increases external validity, the majority of experts were from Europe and only 1 occupational therapist was represented. However, physiotherapists have more basic training in motor assessment of the lower extremity.

The current comprehensive FMA manual for motor assessment was developed in English, which limits its wider use before a broader language translation is completed. In translations the standardized procedures for cultural and linguistic adaptations are required.^23,74,75^ The developed manual will be openly accessible for implementation in clinical practice and research and can preferably be used as a reference point for future adaptations of the FMA scale suitable for digital, instrumented or remote assessment. Finally, implementation of the manual in stroke rehabilitation may serve as a valuable resource for healthcare professionals in developing and evaluating rehabilitation programs.

It is important to note that the FMA focuses primarily on assessment of synergy dependence and ability to perform selected voluntary movements without compensation from other parts of the body, which means that an additional assessment of muscle strength as well as movement quality, using high-fidelity motion capture systems, is warranted.^76^ The direct impact of spasticity to the FMA scores is unknown, although the upper limb spasticity has shown to correlate with the severity of upper limb motor impairment.^77,78^

To better match the multi-center trial designs, further research should evaluate the agreement and accuracy of the current FMA manual when used by multiple raters in multiple sites, provided the proposed recommendations for administration and assessor training are followed. There is also a need to further refine the responsiveness indicators, such as MCID for the FMA in any stage of recovery, and particularly for the FMA-LE by using rigorous methodology. The evidence-base for measurement properties is also limited concerning specific stroke populations, such as, patients with cognitive impairment, bilateral stroke, aphasia, spasticity, neglect and/or pediatric populations. The systematic review of the FMA measurement properties was only conducted in a single database by using core search terms, which is a limitation. However, the conducted search was considered to be sufficient in the context of the current study as majority of the literature was already known to the expert group.

## Conclusions

The development of this consensus-based manual represents a significant advancement in standardizing the training and administration of the Fugl-Meyer Assessment globally. The detailed unified testing procedures will enhance the consistency and reliability of administering the FMA across diverse clinical and research settings, thereby facilitating the comparability of data in multicenter stroke trials focused on motor function. Unified assessment procedures can improve precision, responsiveness and interpretability indicators, such as MCID. In a global perspective, implementation of standardized testing can minimize variations between datasets and enable data pooling with higher statistical power.

## Supplemental Material

sj-docx-1-nnr-10.1177_15459683251412300 – Supplemental material for Standardized International Manual of the Fugl-Meyer Assessment of Motor Function After Stroke Supplemental material, sj-docx-1-nnr-10.1177_15459683251412300 for Standardized International Manual of the Fugl-Meyer Assessment of Motor Function After Stroke by Julie Hervé-Colas, Sarah P. Newton, Stefan T. Engelter, Kathryn S. Hayward, Jeremia P.O. Held, Nadine Intering, Gert Kwakkel, Johannes Pohl, Darcy S. Reisman, Anne Schwarz, Katharina S. Sunnerhagen, Janne Marieke Veerbeek, Karin Wiesner, Sarah B. Zandvliet and Margit Alt Murphy in Neurorehabilitation and Neural Repair

sj-pdf-2-nnr-10.1177_15459683251412300 – Supplemental material for Standardized International Manual of the Fugl-Meyer Assessment of Motor Function After Stroke Supplemental material, sj-pdf-2-nnr-10.1177_15459683251412300 for Standardized International Manual of the Fugl-Meyer Assessment of Motor Function After Stroke by Julie Hervé-Colas, Sarah P. Newton, Stefan T. Engelter, Kathryn S. Hayward, Jeremia P.O. Held, Nadine Intering, Gert Kwakkel, Johannes Pohl, Darcy S. Reisman, Anne Schwarz, Katharina S. Sunnerhagen, Janne Marieke Veerbeek, Karin Wiesner, Sarah B. Zandvliet and Margit Alt Murphy in Neurorehabilitation and Neural Repair

sj-pdf-3-nnr-10.1177_15459683251412300 – Supplemental material for Standardized International Manual of the Fugl-Meyer Assessment of Motor Function After Stroke Supplemental material, sj-pdf-3-nnr-10.1177_15459683251412300 for Standardized International Manual of the Fugl-Meyer Assessment of Motor Function After Stroke by Julie Hervé-Colas, Sarah P. Newton, Stefan T. Engelter, Kathryn S. Hayward, Jeremia P.O. Held, Nadine Intering, Gert Kwakkel, Johannes Pohl, Darcy S. Reisman, Anne Schwarz, Katharina S. Sunnerhagen, Janne Marieke Veerbeek, Karin Wiesner, Sarah B. Zandvliet and Margit Alt Murphy in Neurorehabilitation and Neural Repair
