# Supplementary material for: Standardized International Manual of the Fugl-Meyer Assessment of Motor Function After Stroke
Source: Neurorehabil Neural Repair. 2026 Mar 3;40(4):306–19. doi: 10.1177/15459683251412300 (PMC13005898; doi:10.1177/15459683251412300)
Supplement: sj-docx-1-nnr-10.1177_15459683251412300 – Supplemental material for Standardized International Manual of the Fugl-Meyer Assessment of Motor Function After Stroke [file sj-docx-1-nnr-10.1177_15459683251412300.docx]

Supplemental material 1-5 for article “Standardized international manual of the Fugl-Meyer Assessment of motor function after stroke”, by: Hervé-Colas, Julie; Newton, Sarah P; Engelter, Stefan T; Hayward, Kathryn S; Held, PO Jeremia; Intering, Nadine; Kwakkel, Gert; Pohl Johannes; Reisman, Darcy S; Schwarz Anne; Sunnerhagen, S Katharina; Veerbeek, Janne Marieke; Wiesner, Karin; Zandvliet, Sarah; Alt Murphy, Margit; in Neurorehabilitation and Neural Repair

**Supplemental Table 1.** Modifications and added instructions to the original Fugl-Meyer Assessment for Upper Extremity (Fugl-Meyer et al. 1975) in published manuals of in English.

| **FMA-UE items** | **Modifications and added instructions** | **Reference** |
| --- | --- | --- |
| General instructions | 3 repetition and best of 3 is scored | Sullivan 2011 |
|  | Allows assessment in lying for all items, maximum 3 repetitions and 20 seconds for each item; in case of elbow contracture and 0° starting position not possible, the item is scored as 0; contracture in other than starting position is scored 1 | See 2013 |
| **A. SHOULDER/ELBOW/FOREARM** | | |
| **I. REFLEX ACTIVITY** | | |
| Reflex activity | One of the flexor tendon reflexes is enough to assess | Sullivan 2011, See 2013 |
| **II. MOVEMENTS WITHIN SYNERGIES** | | |
| Flexor Synergy | Starting position: arm on the side | See 2013 |
|  | Starting position: hand resting on the lap, pronated | Deakin 2003 |
| Extensor Synergy |  |  |
| **III. MOVEMENTS WITH MIXED SYNERGIES** | | |
| Hand to lumbar spine | End position: hand higher than anterior superior iliac spine | Deakin 2003 |
| Shoulder flexion 0-90° | Starting position: hand resting on lap | Sullivan 2011 |
|  | Allows assistance to get into starting position | Platz 2005 |
| Pronation-Supination, elbow 90° |  |  |
| **IV. MOVEMENTS WITH LITTLE OR NO SYNERGY DEPENDENCE** | | |
| Shoulder abduction 0-90° | Allows assistance to get into starting position | Platz 2005 |
| Shoulder flexion 90°-180° | Starting position: elbow extended resting on the knee | Sullivan 2011 |
|  | Allows assistance to get into starting position | Platz 2005 |
| Pronation-supination, elbow 0° |  |  |
| **V. NORMAL REFLEX ACTIVITY** | | |
| Normal reflex activity |  |  |
| **B. WRIST** | | |
| Stability at 15° extension, elbow 90° | No support for elbow position is allowed | Sullivan 2011 |
| Repeated extension/flexion, elbow 90° | Requires only 15 degrees extension/flexion to score 2; No support for elbow position is allowed | Sullivan 2011 |
| Stability at 15° extension, elbow 0° | Starting position: shoulder flexed 30°-90° | See 2013 |
|  | Starting position: elbow extended resting on the knee; No support for elbow position is allowed | Sullivan 2011 |
| Repeated extension/flexion, elbow 0° | Starting position: shoulder flexed 30°-90° | See 2013 |
|  | Starting position: elbow extended resting on the knee; Requires only 15 degrees extension/flexion to score 2; No support for elbow position is allowed | Sullivan 2011 |
| Circumduction | Starting position: elbow 0° | See 2013 |
|  | No support for elbow position is allowed | Sullivan 2011 |
|  | Allows support at forearm | Platz 2005 |
| **C. HAND and GRASP** | | |
| Mass flexion/extension | Starting position: arm resting on bedside table  Forearm resting on lap | Sullivan 2011  Page 2012 |
| Hook grasp | Patient grips tester’s finger, resistance not tested | Deakin 2003 |
|  | Starting position: arm resting on bedside table | Sullivan 2011 |
| Thumb abduction | Starting position: arm resting on bedside table; uses lateral grip instead a pure adduction with extended fingers | Sullivan 2011 |
| Pincer grasp | Starting position: arm resting on bedside table; pencil is hold as in writing | Sullivan 2011 |
| Cylinder grasp | Don’t allow contact with the can for finger 3-5 | See 2013 |
|  | Starting position: arm resting on bedside table; patient grasps the cup that is placed on the table | Sullivan 2011 |
|  | Uses can with 8 cm diameter | Deakin 2003 |
|  | Uses a can with approximate 2-3 cm diameter; not cylinder grasp | Platz 2005 |
| Spherical grasp | Starting position: arm resting on bedside table; patient grasps the ball that is placed on the table | Sullivan 2011 |
|  | Grasps the ball only with fingers | See 2013 |
| **D. COORDINATION** | | |
| Coordination/speed | Allows touch to nose with MCP; tremor/dysmetria is scored 2 if patient is unable to move the arm | See 2013 |
|  | Movement is performed only once with eyes open; starting position: hand on lap (not knee); tremor/dysmetria is scored 2 if patient is unable to move the arm and no tremor/dysmetria is observed on face, voice, arms or legs | Sullivan 2011 |
|  | Starting position: shoulder abduction 90°, if this position is not possible scores 0 | Platz 2005 |

**Supplemental Table 2.** Modifications and added instructions the original manual of the Fugl-Meyer Assessment for Lower Extremity (Fugl-Meyer et al. 1975) in published manuals of in English.

| **Items tested** | **Modifications and added instructions** | **Publication** |
| --- | --- | --- |
| General instructions | 3 repetition and best of 3 is scored | Sullivan 2011 |
|  | in case of elbow contracture and 0° starting position not possible, the item is scored as 0; contracture in other than starting position is scored 1 | See 2013 |
| **A. HIP / KNEE / ANKLE** | | |
| **I. REFLEX ACTIVITY** | | |
| Reflex activity | One of the extensor tendon reflexes is enough to assess (no difference in scoring); allows assessment in sitting; | Sullivan 2011 |
| **II. MOVEMENTS WITHIN SYNERGIES** | | |
| Flexor Synergy |  |  |
| Extensor Synergy | Starting position: lying on the side | Sullivan 2011 |
| **III. MOVEMENTS WITH MIXED SYNERGIES** | | |
| Knee flexion beyond 90° |  |  |
| Ankle dorsal flexion |  |  |
| **IV. MOVEMENTS WITH LITTLE OR NO SYNERGY DEPENDENCE** | | |
| Knee flexion, hip 0° |  |  |
| Ankle dorsiflexion | Starting position: allows dorsiflexion with leg moved forward, in case limited ROM in standing | Sullivan 2011 |
| **D. COORDINATION** | | |
| Coordination/speed | Movement is performed eyes open; starting position: heel resting on opposite ankle; heel is moved along the shin bone; scores 0 if active ROM is less than the non-affected side; tremor/dysmetria is scored 2 if patient is unable to move the leg and no tremor/dysmetria is observed on face, voice, arms or legs | Sullivan 2011 |

**References used in Supplemental Table 1 and 2**

Deakin A, Hill H, Pomeroy VM. Rough Guide to the Fugl-Meyer Assessment. Upper limb section. Physiotherapy. 2003;89(12):751-63.

Page SJ, Levine P, Hade E. Psychometric properties and administration of the wrist/hand subscales of the Fugl-Meyer Assessment in minimally impaired upper extremity hemiparesis in stroke. Arch Phys Med Rehabil. 2012;93(12):2373-6 e5.

Platz T, Pinkowski C, van Wijck F. Arm Rehabilitation Measurement: Deutscher Wissenschafts-Verlag (DWV); 2005. 144 p.

See J, Dodakian L, Chou C, Chan V, McKenzie A, Reinkensmeyer DJ, et al. A standardized approach to the Fugl-Meyer assessment and its implications for clinical trials. Neurorehabil Neural Repair. 2013;27(8):732-41.

Sullivan KJ, Tilson JK, Cen SY, Rose DK, Hershberg J, Correa A, et al. Fugl-Meyer assessment of sensorimotor function after stroke: standardized training procedure for clinical practice and clinical trials. Stroke. 2011;42(2):427-32.

**Supplemental Table 3.** The critical points identified and discussed during the Fugl-Meyer Assessment (FMA) manual development process along with the explanation to a consensus-based specifications.

| **General instructions** |
| --- |
| Deviations from correct testing position (e.g. sitting or supine) |
| - It should be documented on the assessment sheet what position was used when it deviates from the standard position. If an alternate position is used, the assessor needs to decide whether correct scoring can be made. If not, the assessor marks the item ‘not testable’ and scores it as 0. - Allowing deviations facilitates assessment in clinical settings. Additional specified instructions can be considered in research settings on a trial-to-trial bases. |
| Number of trials (not defined in the original FMA, although it is emphasized to ensure that the patient understands instructions) |
| - The number of repetitions should be kept low (1-3). Best performance is scored if performance varies between repetitions - Several repetitions are most relevant for scoring flexion and extension synergies or when the patient has difficulties to follow instructions. Note that the number of repetitions is not strictly limited to a specific number. |
| ‘Not testable’ (not defined in the original FMA) |
| - The item is ‘not testable’ when correct assessment can’t be made due to: 1) deviations in test position, 2) other restrictions, such as, amputation, pain, apraxia. The item is scored as 0 and the reason is noted in the assessment sheet. |
| **A. Shoulder/ elbow/forearm** |
| Contracture of elbow joint (item 13,15, 16, 17, 21, 22) (Not specified in the original FMA) |
| - If elbow joint contracture is ≥30°, the items cannot be assessed correctly and are marked as ‘not testable’ and scored as 0. - An extension deficit of up to 30° due to joint contracture (not spasticity) can be considered as patient’s maximum available range of motion |
| Pronation/supination (item 17) |
| - Starting position: shoulder to approximately 30° flexion. - The original description was between 30°-90° degrees, but approximately 30° was used most by experts. Allowing up to 90°shoulder flexion could introduce a bias, due to the longer lever arm and effort needed to keep this position. |
| **B. Wrist** |
| Circumduction (item 23) |
| - Starting position: 90° elbow flexion. - Circumduction of the wrist needs to be a circular motion combining flexion, extension, and radioulnar deviation without excessive simultaneous supination or pronation of the forearm. Note that minor supination/pronation occurs naturally during a smooth and maximal circumduction. - The original paper does not state specifically the elbow position for this item but describes that only 2 items of the wrist section are performed with elbow at 0°, which makes it clear that the elbow should be in 90° of flexion. |
| **C. Hand** |
| Cylinder grasp & spherical grasp (item 29 & 30) |
| - The fingers need to be actively extended in preparation to grasp the can/ball presented close to the hand and fingers. Patient holds the can/ball with opposed thumb and all fingers around the can/ball; the volar surface of the hand is in contact with the can/ball to form a power cylinder/spherical grip. - For cylinder grasp, the original paper is unclear and states that the volar surface of the first and second fingers are against each other. The Swedish original description states that the same fingers grasp around the can. The expert users from when the scale was developed confirm a cylinder grasp with all fingers has been used originally. By definition, in a cylindrical grasp the palmar surface of the hand and fingers are curved around the object with an opposed thumb (observe curved around and that all fingers are used in cylinder grip). - For spherical grasp, the original paper states, fingers in slight abduction/flexion, thumb opposed to grasp around the ball. The expert users from the time the scale was developed confirm that a whole hand spherical grasp with the ball in the hand (not a fingertip grasp) was used originally. By definition, in a spherical grasp, the hand is curved to hold a ball with the palm of the hand in contact with the ball with fingers around the ball (observe the palm of the hand in contact with the object). |
| **E. Lower Extremity** |
| Ankle dorsal flexion (Item 11 &13) |
| - To score 2, full active dorsal flexion of available passive range of motion in sitting/standing position is required - The non-affected side can be used as reference, when it is known that no impairment, joint movement deficit or pain influencing performance exists (general instructions). |
