# Supplementary material for: Standardized International Manual of the Fugl-Meyer Assessment of Motor Function After Stroke
Source: Neurorehabil Neural Repair. 2026 Mar 3;40(4):306–19. doi: 10.1177/15459683251412300 (PMC13005898; doi:10.1177/15459683251412300)
Supplement: sj-pdf-2-nnr-10.1177_15459683251412300 – Supplemental material for Standardized International Manual of the Fugl-Meyer Assessment of Motor Function After Stroke [file sj-pdf-2-nnr-10.1177_15459683251412300.pdf]

Fugl-Meyer Assessment Lower Extremity FMA-LE

Instruction manual

Comprehensive updated and agreed manual of the Fugl-Meyer Assessment of Lower Extremity (FMA-LE) motor function for people with hemiparesis due to stroke.

This manual is based on and follows the original publication by:

Fugl-Meyer AR, Jaasko L, Leyman I, Olsson S, Steglind S. The post-stroke hemiplegic patient. 1. a method for evaluation of physical performance. *Scand J Rehabil Med.* 1975;7(1):13-31.

Contributors
Margit Alt Murphy, Julie Hervé-Colas, Sarah P Newton, Stefan T Engelter, Kathryn S Hayward, Jeremia PO Held, Nadine Intering, Gert Kwakkel, Johannes Pohl, Darcy S Reisman, Anne Schwarz, Katharina S Sunnerhagen, Janne Marieke Veerbeek, Karin Wiesner, Sarah B Zandvliet

The manual can be used for clinical or research purposes, provided that no charge or profit is made for any course or event for which they are used. A reference to the publication should be made when used. For official translations of the manual please contact margit.alt-murphy@neuro.gu.se prior to planned translation.

SCORE Stroke shared photographs used for this manual. Dr Kate Hayward received seed funding for SCORE Stroke from University of Melbourne, Australia. Photographs with SCORE Stroke acknowledgment can be used for education purposes only, provided that no charge or profit is made for any course or event for which they are used. For any other purposes please contact kate.hayward@unimelb.edu.au prior to use.

Instructional videos for how to administer the FMA-LE in accordance with this manual are available at the University of Gothenburg homepage https://www.gu.se/en/neuroscience-physiology/fugl-meyer-assessment.

Correct reference: Hervé-Colas J, Newton SP Engelter ST, Hayward KS, Held PO, Intering N, Pohl J, Reisman DS, Schwarz A, Sunnerhagen SK, Veerbeek JM, Wiesner K, Zandvliet SB, Alt Murphy M. Standardized international manual of the Fugl-Meyer Assessment of motor function after stroke. Neurorehabil Neural Repair. 2026. DOI: 10.1177/15459683251412300

2026

Fugl-Meyer Assessment of Lower Extremity (FMA-LE) Motor Assessment

Item	Page	Item description	Score	Comments
		**E. HIP / KNEE / ANKLE**		
		**I. Reflex activity (max 4)**		
01	5	Flexors	0 2	
02	5	Extensors	0 2	
		**II. Movements within synergies (max 14)**		
03	6	Flexor synergy: Hip flexion	0 1 2	
04		Knee flexion	0 1 2	
05		Ankle dorsal flexion	0 1 2	
06	7	Extensor synergy: Hip extension	0 1 2	
07		Hip adduction	0 1 2	
08		Knee extension	0 1 2	
09		Ankle plantar flexion	0 1 2	
		**III. Movements with mixed synergies (max 4)**		
10	8	Knee flexion beyond 90°, sitting	0 1 2	
11	9	Ankle dorsal flexion, sitting	0 1 2	
		**IV. Movements with little or no synergy dependence (max 4)**		
12	10	Knee flexion, hip 0°, standing	0 1 2	
13	11	Ankle dorsiflexion, standing	0 1 2	
		**V. Normal reflex activity (max 2)**		
14	12	Flexors/extensors	0 1 2	
		Total E (max 28)		
		**F. COORDINATION/SPEED**		
15	13-14	Tremor	0 1 2	
16	13-14	Dysmetria	0 1 2	
17	13-14	Time	0 1 2	
		Total F (max 6)		
		**TOTAL FMA-LE (MAX 34)**		

Fugl-Meyer Assessment - Lower Extremity (FMA-LE) Motor Assessment

General instructions

Position

- The standard positions for rating the items of the FMA-LE are lying, sitting or standing. In a sitting position, either a chair, a wheelchair, a bed or an examination table can be used given that patient's feet are resting on the floor. Balance support is allowed both in sitting and standing.
- When a patient is unable to achieve the standing position, the assessor marks the item 'not testable' and scores 0.
- When the supine position is not possible due to practical reasons, the assessor needs to decide whether correct scoring for these items (reflexes, flexion/extension synergy, coordination/speed) can be made in sitting position. It should be documented on the assessment sheet what position was used when it deviates from the standard position. If correct scoring can't be made, the assessor marks the item 'not testable' and scores it as 0.

Instructions and assistance

- Each movement is explained and demonstrated by the assessor.
- Physical guidance to demonstrate the correct movement can be used to ensure the patient's understanding (for example in the presence of aphasia, apraxia or neglect).
- The patient can be asked to repeat the movement if the patient doesn't follow the instructions correctly or when the assessor needs to see a specific part of the movement.
- The number of repetitions should be kept low (1-3). Best performance is scored if performance varies between attempts.
- The patient can be asked to perform each movement with the non-affected leg first to ensure that the patient understands the instructions and to compare to the non-affected side if relevant.
- In general, no assistance is provided for active movements of the tested body parts, neither by the assessor or the patient themselves. Details on when assistance and/or support are allowed is specified under each item and in the 'Quick guide on assistance and support allowed' (Page 15).

Scoring

- All items are scored on a scale of 0 to 2. The detailed instructions for scoring are specified under each item.
- Compensatory movements with other body parts (e.g., trunk and hip) are not allowed.
- A lower score should be selected if uncertainty exists between the scoring levels.
- The order of the items can be changed. For example, the reflex items can be performed at the end.
- The performance of the non-affected leg can be used as a reference in scoring, when it is known that no impairment, joint movement deficit or pain influencing performance exists.
- The assessor needs to test the passive range of motion before scoring items that require full active joint range of motion to receive a maximum score.
- If the passive range of motion is markedly limited due to joint contracture (restriction exceeding ¼ of the normal joint range), full voluntary movement of that joint cannot be assessed. The item is scored as 1 or 0 (according to the specific requirements of the item) and the reason is noted on the patient's assessment sheet.
- If a specific movement cannot be performed due to other problems, such as amputation, pain, or apraxia the item is 'not testable'. The item is scored 0 and the reason is noted in the assessment sheet.
- If spasticity is present, use slow stretch of the muscles to test the passive range of motion before scoring.

Materials

- ☑ Bed or examination table 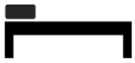
- ☑ Chair with or without armrests 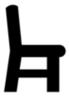
- ☑ Tendon reflex hammer 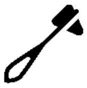
- ☑ Stopwatch 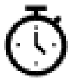

E. Lower extremity

E. I. Reflex activity

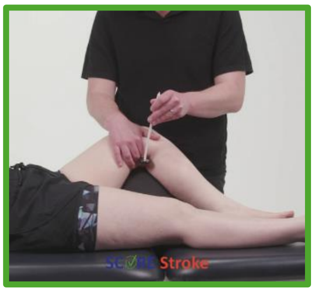

FLEXORS – Knee
Start position

- ☑ Supine position
- ☑ Knee flexed between 30-90°
- ☑ Hip slightly flexed abducted and externally rotated

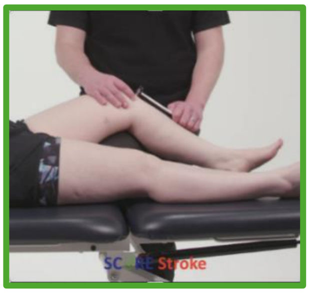

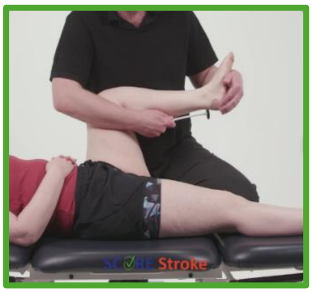

EXTENSORS – Knee (patella) or ankle (Achilles)
Start position

- ☑ Supine position
- ☑ Knee flexed between 30-90°
- ☑ Hip slightly flexed, abducted and externally rotated

Verbal instructions with concurrent physical demonstration

"Relax your leg, I will support it and test your reflexes."

Flexors: The leg is supported by the assessor and the assessor's fingers are placed over the medial knee flexor tendon; a slight distinct tap is applied over the assessor's own fingers to elicit a reflex response.

Extensors: The leg is supported by the assessor; a slight distinct tap is applied on the patella tendon to elicit a reflex response. For the Achilles tendon reflex the assessor holds the patient's foot in slight dorsal extension and applies a slight distinct tap on the Achilles tendon to elicit reflex response.

Points for consideration

- Reflex activity of the non-affected side is tested first and used for comparison
- The patient can be instructed to contract the muscle shortly before tapping the tendon to help them relax
- Reflexes can be verified either visually or by palpation of the tendon
- Other methods of testing reflexes, in addition to those described above, are accepted
- There is no score of 1 for either reflex item
- To score 2, reflex activity of one of the extensor tendons (either Patella or Achilles tendon) needs to be elicited

Scoring

Flexors **ITEM 01**

**0**	No reflex activity
**2**	Reflex activity can be elicited

Extensors **ITEM 02**

**0**	No reflex activity
**2**	Reflex activity can be elicited

E. II. Movements within synergies

FLEXOR SYNERGY, supine position

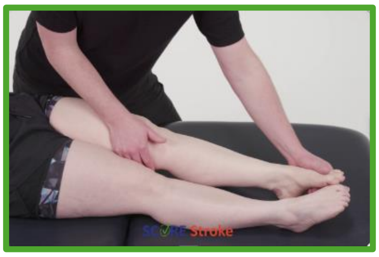

Start position (same as the end position of the extensor synergy)

- ☑ Hip adducted (foot against the non-affected foot)
- ☑ Knee fully extended
- ☑ Full ankle plantar flexion or foot in relaxed position

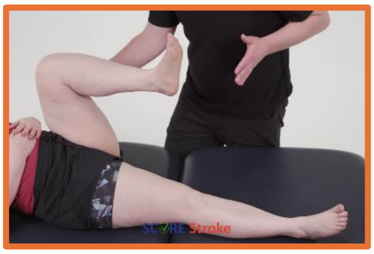

End position

- ☑ Hip and knee fully flexed
- ☑ Full ankle dorsal flexion

Verbal instructions with concurrent physical demonstration

> *"Raise and bend your knee fully toward your chest, also bend your ankle up towards your head."*

Assistance can be provided to move the leg into the start position or as close to this position as possible. No support is allowed during an active movement attempt.

Points for consideration

- Each component is scored separately and must be performed within the movement synergy
- Performance can be compared to the non-affected side
- The active movement is scored from the start position
- Hip flexion can occur in combination with hip abduction and external rotation
- The distal tendons of the knee flexors should be palpated to ensure that knee flexion is active
- To enable observation of each component the assessor can ask the patient to repeat the synergy movement, but the number of repetitions should be kept low (1 to 3)

Scoring

Hip flexion **ITEM 03**

**0**	No hip flexion
**1**	Performs only partially, any degree of hip flexion less than full passive range
**2**	Full hip flexion

Knee flexion **ITEM 04**

**0**	No knee flexion
**1**	Performs only partially, any degree of knee flexion less than full passive range
**2**	Full knee flexion

Ankle dorsiflexion **ITEM 05**

**0**	No ankle dorsal flexion
**1**	Performs only partially, any degree of ankle dorsal flexion less than full passive range
**2**	Full ankle dorsal flexion

EXTENSOR SYNERGY, supine position

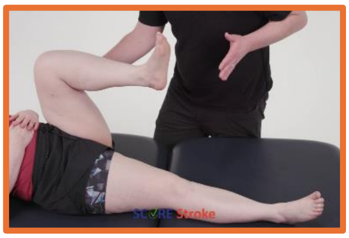

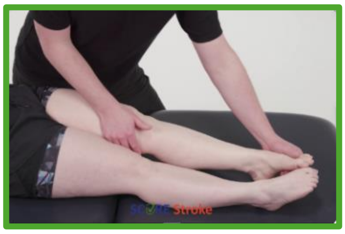

Start position (same as the end position of the flexion synergy)

- ☑ Hip and knee fully flexed
- ☑ Full ankle dorsal flexion

End position

- ☑ Hip adducted (foot against the non-affected foot)
- ☑ Knee fully extended
- ☑ Full ankle plantar flexion

Verbal instructions with concurrent physical demonstration

“Straighten your whole leg down onto the bed close to the other leg and press your foot and toes down.”

Assistance can be provided to move the leg into the start position or as close to this position as possible. Resistance is provided to each movement component by the assessor to eliminate gravitational assistance and ensure that the movement is active.

Points for consideration

- Each component is scored separately and must be performed within the movement synergy
- Performance can be compared to the non-affected side
- The active movement is scored from the start position
- To enable observation of each component the assessor can ask the patient to repeat the synergy movement, the number of repetitions should be kept low (1 to 3)

Scoring

Hip extension **ITEM 06**

0	No active hip extension
1	Performs only partially **OR**<br>Weaker hip extension against resistance compared to the non-affected leg
2	Full hip extension in supine position and strength against resistance comparable to the non-affected leg

Hip adduction **ITEM 07**

**0**	No active hip adduction
**1**	Performs only partially **OR**<br>Weaker hip adduction against resistance compared to the non-affected leg
**2**	Full adduction to the midline and strength against resistance comparable to the non-affected leg

Knee extension **ITEM 08**

**0**	No active knee extension
**1**	Performs only partially **OR**<br>Weaker knee extension against resistance compared to the non-affected leg
**2**	Full extension and strength against resistance comparable to the non- affected leg

Ankle plantar flexion **ITEM 09**

**0**	No active ankle plantar flexion
**1**	Performs only partially **OR**<br>Weaker ankle plantar flexion against resistance compared to the non-affected leg
**2**	Full ankle plantar flexion and strength against resistance comparable to the non-affected leg

E. III. Movements with mixed synergies

KNEE FLEXION BEYOND 90°, sitting

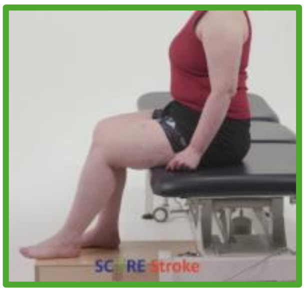

Start position

☑ Knee slightly extended past 90°, foot resting on the floor

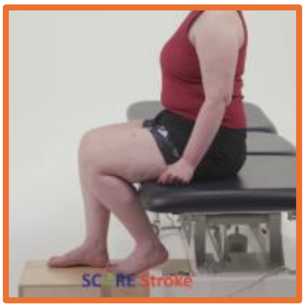

End position

☑ Knee flexed beyond 90°

Verbal instructions with concurrent physical demonstration

"Straighten your knee slightly and then bend your knee as far as possible under the chair/bed."

Assistance can be provided to extend the knee slightly into the starting position; no support is allowed during the active movement.

Points for consideration

- Ensure that the seat or examination table is not restricting the knee flexion
- Do not allow compensatory movement of the trunk or hip
- To score 1, the distal tendons of the knee flexors can be palpated to ensure that knee flexion is active

Scoring **ITEM 10**

**0**	No active knee flexion
**1**	Knee can actively be flexed from slight extended position but not beyond 90°
**2**	Knee flexion beyond 90°

ANKLE DORSAL FLEXION, sitting

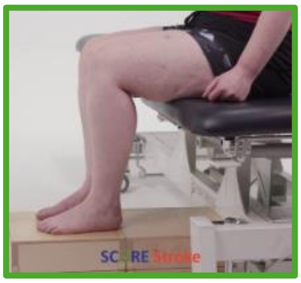

Start position

- ☑ Knee flexed to approximately 90°
- ☑ Foot flat on the floor

End position

- ☑ Full ankle dorsal flexion

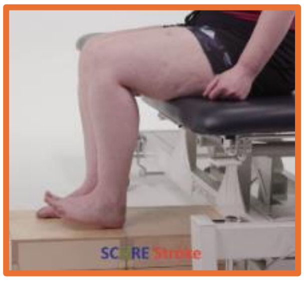

Verbal instructions with concurrent physical demonstration

“Bend your ankle upward as far as you can; keep your heel on the floor.”

Assistance: No support is allowed

Points for consideration

- Ankle movement is preferably assessed without shoes
- To score 2, full active dorsal flexion of available passive range of motion in sitting position is required

Scoring **ITEM 11**

**0**	No active ankle dorsal flexion
**1**	Performs only partially, any degree of ankle dorsal flexion less than full passive range
**2**	Full ankle dorsal flexion

E. IV. Movements with little or no synergy dependence

KNEE FLEXION TO 90°, standing

Start position

- ☑ Hip in neutral position at 0°
- ☑ Knee fully extended

End position

- ☑ Hip in neutral position at 0°
- ☑ Knee flexed to at least 90°

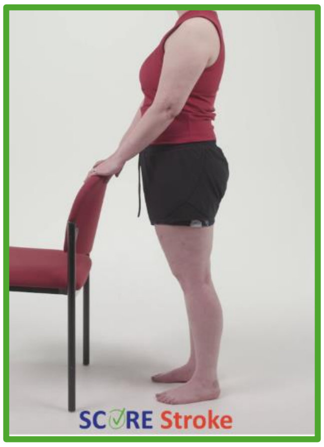

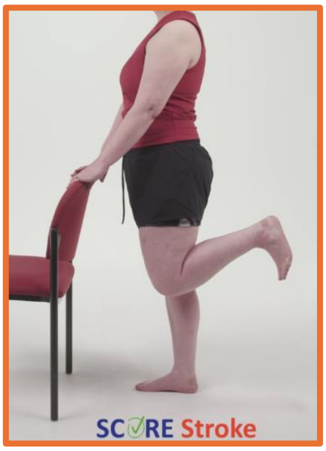

Verbal instructions with concurrent physical demonstration

"Bend your knee behind you while you keep your hip and trunk straight."

Assistance: Balance support is allowed

Points for consideration

- Do not allow compensatory hip and/or trunk movements
- Note, that a slight lateral shift of the bodyweight towards the non-affected leg before the knee flexion is accepted
- When the patient can't extend the hip fully to 0°, the maximum extension achieved in standing position is noted by the assessor; this hip position needs to be kept stable during the knee movement to score 1 or 2

Scoring **ITEM 12**

**0**	No knee flexion **OR**<br>Cannot actively maintain the start position with a fully extended hip when knee flexion begins (hip is flexed immediately)
**1**	Performs only partially, any degree of knee flexion less than 90° **OR**<br>Maintains hip extension in the beginning of the movement, but hip flexion occurs before reaching 90° knee flexion
**2**	Knee flexion to 90° while maintaining full hip extension

ANKLE DORSAL FLEXION, standing

Start position

- ☑ Hip in neutral position at 0°
- ☑ Knee fully extended

End position

- ☑ Hip in neutral position at 0°
- ☑ Knee fully extended
- ☑ Full ankle dorsal flexion

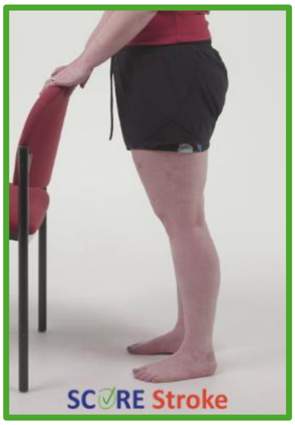

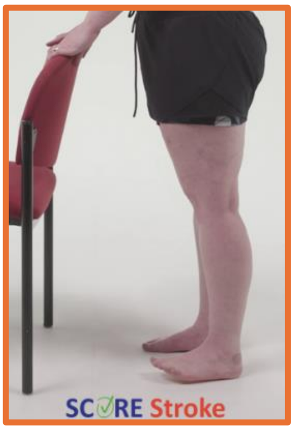

Verbal instructions with concurrent physical demonstration

"Bend your ankle to bring your toes up as far as you can while keeping your heel on the floor and your hip and trunk straight."

Assistance: Balance support is allowed

Points for consideration

- Ankle movement is preferably assessed without shoes
- Do not allow compensatory trunk movements
- Note, that a slight lateral shift of the bodyweight towards the non-affected leg before the ankle dorsal flexion is accepted
- When the patient can't extend the hip fully to 0°, the maximum extension achieved in standing position is noted by the assessor; this hip position needs to be kept stable during the ankle movement to score 1 or 2.
- To score 2, full active dorsal flexion of available passive range of motion in standing position is required

Scoring

ITEM 13

**0**	No ankle dorsal flexion **OR**<br>Cannot actively maintain the start position with a fully extended hip when ankle dorsal flexion begins (hip is flexed immediately)
**1**	Performs only partially, any degree of ankle dorsal flexion less than full passive range **OR**<br>Maintains hip extension in the beginning of the movement, but hip flexion occurs before reaching full ankle dorsal flexion
**2**	Full ankle dorsal flexion while maintaining full hip extension

E.V. Normal reflex activity, supine

- ☑ Assessed only if full score of 4 points was achieved in the previous section E. IV. Movements with little or no synergy dependence
- ☑ If score < 4 in section E. IV, the score in this section is marked as 0
- ☑ The reflexes are scored in the same way as described in E. I. Reflex activity (Page 5)
- ☑ Reflex activity is tested in flexors (knee flexors) and extensors (Patellar and Achilles)

Points for consideration

- Test reflex on the non-affected side first; to use as a comparison
- The patient can be instructed to contract the muscle shortly before tapping the tendon to help them relax
- The reflexes can be verified either visually or by palpation of the tendon
- A markedly hyperactive reflex is when the response is very easily or almost spontaneously elicited OR when the response is markedly stronger compared to the non-affected side
- Lively reflex response can be considered as a lower grade response compared to 'markedly hyperactive' or compared to the non-affected side

Scoring **ITEM 14**

**0**	At least 2 of the 3 reflexes are markedly hyperactive **OR**<br>Scored less than 4 points in part IV
**1**	1 reflex markedly hyperactive **OR**<br>At least 2 reflexes lively
**2**	Maximum of 1 reflex lively and none markedly hyperactive

F. Coordination – speed, supine

- ☑ The heel to knee-cap test needs to be completed 5 times with eyes closed or blindfolded
- ☑ Confirm that the patient can lift the affected leg and touch their heel to their knee-cap
- ☑ The heel to knee-cap movement cycle needs to be completed 5 times, if not, all three items are scored 0
- ☑ A complete heel to knee-cap movement: 1) starts with the leg resting on the bed, 2) the tested leg moves to touch the knee-cap with the heel, 3) the movement ends when the leg is back in the starting position on the bed
- ☑ Use practice trials to ensure that the patient understood the instructions
- ☑ If needed the assessor can count the 5 repetitions out loud
- ☑ Begin timing when the leg leaves the bed and stop when the leg is back on the bed after 5 repetitions
- ☑ Record the time for both legs by timing the non-affected leg first; the difference in time between the legs is used in scoring

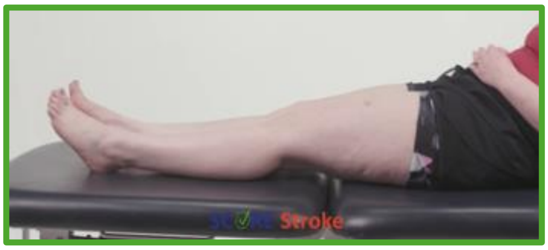

Start and end position

- ☑ Leg is resting on the bed
- ☑ Eyes closed or blindfolded

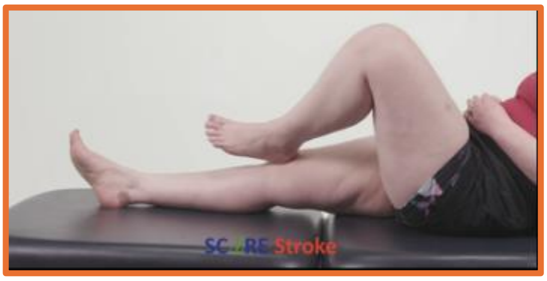

Heel on the knee-cap position

- ☑ Heel touches the knee-cap

Verbal instructions with concurrent physical demonstration

"Bring your heel to the knee-cap of the other leg five times, as fast and precisely as possible. I am going to take time. Keep your eyes closed."

Assistance: Not allowed

Points for consideration

- **Tremor** is assessed as any deviation from a straight and smooth movement trajectory[1]
- **Dysmetria** is assessed as any deviation from the heel touching the middle part of the knee-cap
  - **Slight dysmetria** is scored when the heel touches a close circle around the knee-cap
  - **Pronounced dysmetria** is scored when the heel touch is further away from the knee given that the patient can touch the knee when movement is performed slowly
- Score 2 for dysmetria if only first movement shows slight deviation from the knee-cap; and all following 4 movements point correctly on the knee-cap
- Score 0 if the dysmetria is either pronounced or unsystematic

---

[1] Rodrigues MR, Slimovitch M, Chilingaryan G, Levin MF. Does the Finger-to-Nose Test measure upper limb coordination in chronic stroke? J Neuroeng Rehabil. 2017 Jan 23;14(1):6.

Scoring

Tremor **ITEM 15**

**0**	Marked tremor that substantially interferes with coordination **OR**<br>Unable to complete 5 repetitions of the task
**1**	Slight tremor, that mildly interferes with coordination
**2**	No tremor is observed during all 5 repetitions

Dysmetria **ITEM 16**

**0**	Pronounced or unsystematic dysmetria (touches randomly on different locations away from the knee) **OR**<br>Unable to complete 5 repetitions of the task
**1**	Slight but systematic dysmetria (touches systematically in the same location close to the knee-cap)
**2**	No dysmetria (the heel lands on the knee-cap during all 5 repetitions)

Time **ITEM 17**

**0**	The affected leg is 6 or more seconds slower than the non-affected leg **OR**<br>Unable to complete 5 repetitions of the task
**1**	The affected leg is 2.0 to 5.9 seconds slower than the non-affected leg
**2**	The affected leg is less than 2 seconds slower (or faster) than the non-affected leg

Appendix. Quick guide on assistance and support allowed

Items tested	Assistance during active movement	Details on assistance and support
**E. HIP / KNEE / ANKLE**		
**II. MOVEMENTS WITHIN SYNERGIES**		
Flexor synergy	**NO**	Only allowed to move the leg into the start position.
Extensor synergy	**NO**	Only allowed to move the leg into the start position. During the active synergy movement antigravitational resistance is applied to ensure that the movement is active.
**III. MOVEMENTS WITH MIXED SYNERGIES**		
Knee flexion beyond 90°, sitting	**NO**	Only allowed to move the leg into the start position. Balance support is allowed during movement.
Ankle dorsal flexion, sitting	**NO**	Full passive range of ankle dorsal flexion is assessed before scoring in sitting. Balance support is allowed during movement.
**IV. MOVEMENTS WITH LITTLE OR NO SYNERGY DEPENDENCE**		
Knee flexion, standing	**NO**	Balance support is allowed during movement.
Ankle dorsal flexion, standing	**NO**	Full passive range of ankle dorsal flexion is assessed before scoring in standing. Balance support is allowed during movement.
**F. COORDINATION / SPEED**		
Coordination/speed	**NO**	No support allowed
