# Supplementary material for: Standardized International Manual of the Fugl-Meyer Assessment of Motor Function After Stroke
Source: Neurorehabil Neural Repair. 2026 Mar 3;40(4):306–19. doi: 10.1177/15459683251412300 (PMC13005898; doi:10.1177/15459683251412300)
Supplement: sj-pdf-3-nnr-10.1177_15459683251412300 – Supplemental material for Standardized International Manual of the Fugl-Meyer Assessment of Motor Function After Stroke [file sj-pdf-3-nnr-10.1177_15459683251412300.pdf]

Fugl-Meyer Assessment Upper Extremity FMA-UE

Instruction manual

Comprehensive, updated and agreed manual of the Fugl-Meyer Assessment of Upper Extremity (FMA-UE) motor function for people with hemiparesis due to stroke.

This manual is based on and follows the original publication by:

Fugl-Meyer AR, Jaasko L, Leyman I, Olsson S, Steglind S. The post-stroke hemiplegic patient. 1. a method for evaluation of physical performance. *Scand J Rehabil Med.* 1975;7(1):13-31.

Contributors
Margit Alt Murphy, Julie Hervé-Colas, Sarah P Newton, Stefan T Engelter, Kathryn S Hayward, Jeremia PO Held, Nadine Intering, Gert Kwakkel, Johannes Pohl, Darcy S Reisman, Anne Schwarz, Katharina S Sunnerhagen, Janne Marieke Veerbeek, Karin Wiesner, Sarah B Zandvliet

The manual can be used for clinical or research purposes, provided that no charge or profit is made for any course or event for which they are used. A reference to the publication should be made when used. For official translations of the manual please contact margit.alt-murphy@neuro.gu.se prior to planned translation.

SCORE Stroke shared photographs used for this manual. Dr Kate Hayward received seed funding for SCORE Stroke from University of Melbourne, Australia. Photographs with SCORE Stroke acknowledgment can be used for education purposes only, provided that no charge or profit is made for any course or event for which they are used. For any other purposes please contact kate.hayward@unimelb.edu.au prior to use.

Instructional videos for how to administer the FMA-UE in accordance with this manual are available at the University of Gothenburg homepage https://www.gu.se/en/neuroscience-physiology/fugl-meyer-assessment.

Correct reference: Hervé-Colas J, Newton SP Engelter ST, Hayward KS, Held PO, Intering N, Pohl J, Reisman DS, Schwarz A, Sunnerhagen SK, Veerbeek JM, Wiesner K, Zandvliet SB, Alt Murphy M. Standardized international manual of the Fugl-Meyer Assessment of motor function after stroke. Neurorehabil Neural Repair. 2026. DOI: 10.1177/15459683251412300

2026

Fugl-Meyer Assessment of Upper Extremity (FMA-UE) Motor Assessment

Item	Page	Item description	Score	Comments
		**A. SHOULDER/ELBOW/FOREARM**		
		**I. Reflex activity (max 4)**		
01	5	Flexors	0 2	
02	5	Extensors	0 2	
		**II. Movements within synergies (max 18)**		
03	6-7	Flexor synergy: Retraction	0 1 2	
04	6-7	Elevation	0 1 2	
05	6-7	Abduction 90°	0 1 2	
06	6-7	External rotation	0 1 2	
07	6-7	Elbow flexion	0 1 2	
08	6-7	Forearm supination	0 1 2	
09	8-9	Extensor synergy: Adduction/internal rotation	0 1 2	
10	8-9	Elbow extension	0 1 2	
11	8-9	Forearm pronation	0 1 2	
		**III. Movements with mixed synergies (max 6)**		
12	10	Hand to lumbar spine	0 1 2	
13	11	Shoulder flexion 0°-90°, elbow 0°	0 1 2	
14	12	Pronation-supination, elbow 90°	0 1 2	
		**IV. Movements with little or no synergy dependence (max 6)**		
15	13	Shoulder abduction 0°-90°, elbow 0°	0 1 2	
16	14	Shoulder flexion 90°-180°, elbow 0°	0 1 2	
17	15	Pronation-supination, elbow 0°	0 1 2	
		**V. Normal reflex activity (max 2)**		
18	16	Flexors/extensors	0 1 2	
		Total A (max 36)		
		**B. WRIST**		
19	17	Wrist stability at 15° dorsal extension, elbow 90°	0 1 2	
20	18	Repeated wrist extension and flexion, elbow 90°	0 1 2	
21	19	Wrist stability at 15° dorsal extension, elbow 0°	0 1 2	
22	20	Repeated wrist extension and flexion, elbow 0°	0 1 2	
23	21	Circumduction, elbow 90°	0 1 2	
		Total B (max 10)		
		**C. HAND**		
24	22	Mass flexion	0 1 2	
25	23	Mass extension	0 1 2	
26	24	Hook grasp	0 1 2	
27	25	Thumb adduction	0 1 2	
28	26	Pincer grasp	0 1 2	
29	27	Cylinder grasp	0 1 2	
30	28	Spherical grasp	0 1 2	
		Total C (max 14)		
		**D. COORDINATION/SPEED**		
31	29-30	Tremor	0 1 2	
32	29-30	Dysmetria	0 1 2	
33	29-30	Time	0 1 2	
		Total D (max 6)		
		**TOTAL FMA-UE (max 66)**		

Fugl-Meyer Assessment - Upper Extremity (FMA-UE) Motor Assessment

General instructions

Position

- The standard position for rating all items of the FMA-UE is sitting on a chair without armrests.
- When a patient is unable to achieve the standard sitting position, scores can be estimated while sitting in an armchair, wheelchair or in bed with an elevated backrest. It should be documented on the assessment sheet what position was used when it deviates from the standard position. If an alternate position is used, the assessor needs to decide whether correct scoring can be made. If not, the assessor marks the item 'not testable' and scores it as 0.

Instructions and assistance

- Each movement is explained and demonstrated by the assessor, while sitting in front of the patient.
- Physical guidance to demonstrate the correct movement can be used to ensure the patient's understanding (for example in the presence of aphasia, apraxia or neglect).
- The patient can be asked to repeat the movement if the patient doesn't follow the instructions correctly or when the assessor needs to see a specific part of the movement.
- The number of repetitions should be kept low (1-3). Best performance is scored if performance varies between attempts.
- The patient can be asked to perform each movement with the non-affected arm first to ensure that the patient understands the instructions and to compare to the non-affected arm if relevant.
- In general, no assistance is provided for active movements of the tested body parts, neither by the assessor or the patient themselves. Details on when assistance and/or support are allowed is specified under each item and in the 'Quick guide on assistance and support allowed' (Page 31).

Scoring

- All items are scored on a scale of 0 to 2. The detailed instructions for scoring are specified under each item.
- Compensatory movements with other body parts (e.g., trunk) are not allowed.
- A lower score should be selected if uncertainty exists between the scoring levels.
- The order of the items can be changed. For example, the reflex items can be performed at the end.
- The performance of the non-affected arm can be used as a reference in scoring, when it is known that no impairment, joint movement deficit or pain influencing performance exists.
- The assessor needs to test the passive range of motion before scoring items that require full active joint range of motion to receive a maximum score.
- If the passive range of motion is markedly limited due to joint contracture (restriction exceeding ¼ of the normal joint range), full voluntary movement of that joint cannot be assessed. The item is scored as 1 or 0 (according to the specific requirements of the item) and the reason is noted on the patient's assessment sheet.
- If a specific movement cannot be performed due to other problems, such as amputation, pain, or apraxia the item is 'not testable'. The item is scored 0 and the reason is noted in the assessment sheet.
- If spasticity is present, use slow stretch of the muscles to test the passive range of motion before scoring.

Materials

- [x] Chair without armrest
- [x] Tendon reflex hammer
- [x] Pencil
- [x] Sheet of paper (A5 size)
- [x] Small cylindrical can (a hard-plastic empty container with a diameter of approximately 5 cm and maximum weight of 100 g)
- [x] Tennis ball
- [x] Stopwatch

A. Shoulder, elbow, forearm

A. I. Reflex activity

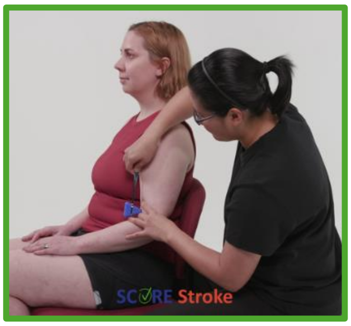

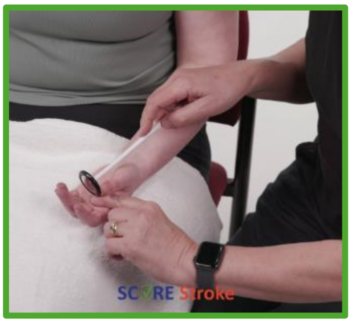

FLEXORS – Elbow (Biceps) and/or fingers

Start position

- ☑ Sitting
- ☑ Hand resting in lap
- ☑ Forearm supinated

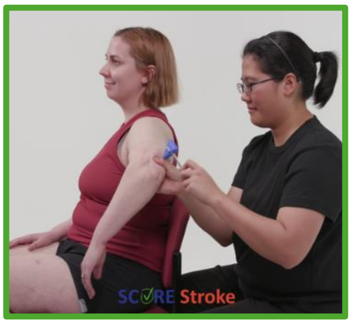

EXTENSORS – Elbow (Triceps)

Start position

- ☑ Sitting
- ☑ Shoulder slightly abducted and internally rotated
- ☑ Forearm relaxed in line with gravity

Verbal instructions with concurrent physical demonstration

"Relax your arm, I will support it and test your reflexes."

Flexors: The arm is stabilised with the assessor's finger/thumb placed on the Biceps tendon; the assessor applies a slight distinct tap on their own finger/thumb to elicit a reflex response.

If the Biceps reflex is difficult to elicit, the finger flexor reflex should be tested. Here, the assessor's index finger is placed across the volar side of the metacarpophalangeal (MCP) joints, a slight distinct tap is applied on the assessor own finger to elicit the reflex response.

Extensors: The arm is stabilised by the assessor in the start position and a slight distinct tap is applied on the triceps tendon, close to the elbow, to elicit a reflex response.

Points for consideration

- Reflex activity of the non-affected arm is tested first and used for comparison
- The patient can be instructed to contract the muscle shortly before tapping the tendon to help them relax
- Reflexes can be verified either visually or by palpation of the tendon
- Other methods of testing reflexes, in addition to those described above, are accepted
- There is no score of 1 for either reflex item
- To score 2, reflex activity of one of the flexors (biceps or finger) needs to be elicited

Scoring

Flexors **ITEM 01**

**0**	No reflex activity
**2**	Reflex activity can be elicited

Extensors **ITEM 02**

**0**	No reflex activity
**2**	Reflex activity can be elicited

A. II. Movements within synergies

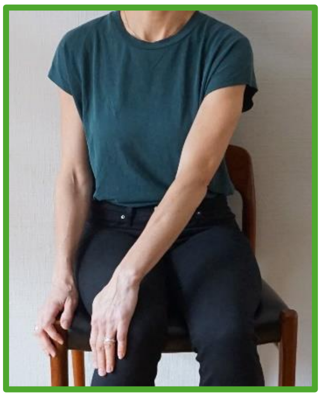

FLEXOR SYNERGY

Start position (same as the end positions of the extensor synergy)

- ☑ Shoulder adducted, protracted and depressed enough to reach the opposite knee
- ☑ Elbow extended to 0°
- ☑ Forearm pronated
- ☑ Hand outside of the contralateral knee

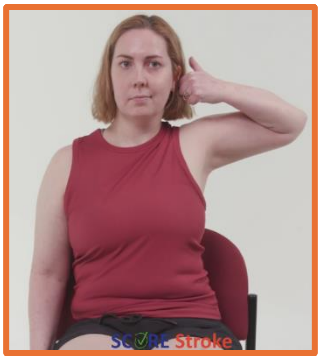

End position

- ☑ Shoulder girdle elevated and retracted, enough to reach the ipsilateral ear
- ☑ Shoulder abducted to 90°
- ☑ Full external rotation, elbow flexion and forearm supination
- ☑ Hand close to the ipsilateral ear, palm facing backward, thumb upwards

Verbal instructions with concurrent physical demonstration

"Put your hand on the outside of your opposite knee. Now bring your hand toward your ear with your elbow at shoulder level, with your palm facing backward (thumb up)."

Assistance can be provided to move the arm to the start position or as close to this position as possible. No support is allowed during an active movement attempt.

Points for consideration

- Each component is scored separately and must be performed within the movement synergy
- Performance can be compared to the non-affected side
- The active movement is scored from the start position; for example, for shoulder elevation/retraction, movement starts from a depressed and protracted shoulder position
- To score 2 for shoulder abduction the end position needs to reach greater than or equal to 90 degrees; it does not need to be the maximum range of motion
- To score 2 for shoulder external rotation and elbow flexion almost full active movement is required to reach the end position (hand touching the ear)
- To score 2 for supination, full supination within available passive range of motion is required
- To enable observation of each component the assessor can ask the patient to repeat the synergy movement, the number of repetitions should be kept low (1 to 3)
- Do not allow flexion or rotation of the trunk and head to compensate for arm movements

Scoring

Retraction of the shoulder girdle **ITEM 03**

**0**	No retraction
**1**	Performs only partially
**2**	Retraction to a degree that is equal to or greater than the end position on the non-affected side

Elevation of the shoulder girdle **ITEM 04**

**0**	No elevation
**1**	Performs only partially
**2**	Elevation to a degree that is equal to or greater than the end position on the non-affected side

Abduction of the shoulder **ITEM 05**

**0**	No abduction
**1**	Performs only partially, any degree of abduction that is less than 90°
**2**	Shoulder abduction to 90°

External rotation **ITEM 06**

**0**	No external rotation
**1**	Performs only partially, any degree of external rotation less than full passive range
**2**	Full external rotation with hand touching the ear

Elbow flexion **ITEM 07**

**0**	No elbow flexion
**1**	Performs only partially, any degree of elbow flexion less than full passive range
**2**	Full elbow flexion with hand touching the ear

Forearm supination **ITEM 08**

**0**	No supination
**1**	Performs only partially, any degree of supination less than full passive range
**2**	Full supination

EXTENSOR SYNERGY

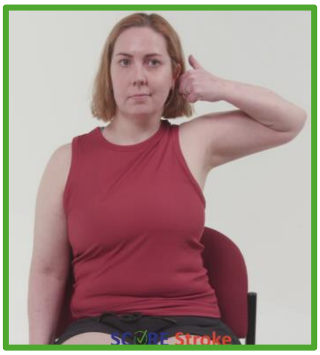

Start position (same as the end position of the flexion synergy)

- ☑ Shoulder girdle elevated and retracted
- ☑ Shoulder abducted 90° degrees
- ☑ Full shoulder external rotation, elbow flexion and forearm supination
- ☑ Hand close to the ipsilateral ear, palm facing backward, thumb upwards

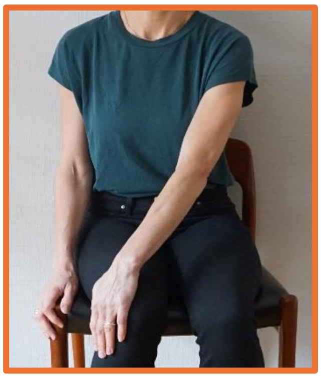

End position

- ☑ Shoulder adducted protracted and depressed, enough to reach the opposite knee
- ☑ Elbow fully extended to 0°
- ☑ Forearm pronated (to position the palm of the hand on the lateral side of the knee)
- ☑ Hand outside of the contralateral knee

Verbal instructions with concurrent physical demonstration

"Hold your hand toward your ear with your elbow at shoulder level, with your palm facing backward (thumb up). Now bring your hand to the outside of your opposite knee and straighten your elbow fully."

Assistance can be provided to move the arm into the start position or as close to this position as possible. No support is allowed during active movement. If required, slight resistance can be provided during the movement to eliminate gravity assistance and ensure that the movement is active.

Points for consideration

- Each component is scored separately and must be performed within the movement synergy
- Performance can be compared to the non-affected side
- The active movement is scored from the start position
- To score 2 for elbow extension, full active elbow extension within available passive range of motion is required to reach the end position
- To score 2 for shoulder adduction/internal rotation and forearm pronation, range needs to be sufficient to reach the end position
- To enable observation of each component the assessor can ask the patient to repeat the synergy movement, the number of repetitions should be kept low (1 to 3)
- Do not allow flexion or rotation of the trunk to compensate for arm movements

Scoring

Adduction/internal rotation of the shoulder **ITEM 09**

0	No adduction/internal rotation
1	Performs only partially
2	Adduction/internal rotation across the midline to the degree that is required to reach the end position

Elbow extension **ITEM 10**

**0**	No elbow extension
**1**	Performs only partially
**2**	Full elbow extension to 0°

Forearm pronation **ITEM 11**

**0**	No pronation
**1**	Performs only partially
**2**	Pronation to a degree that is required to reach the end position with palm of the hand outside the contralateral knee

A. III. Movements with mixed synergies

HAND TO LUMBAR SPINE

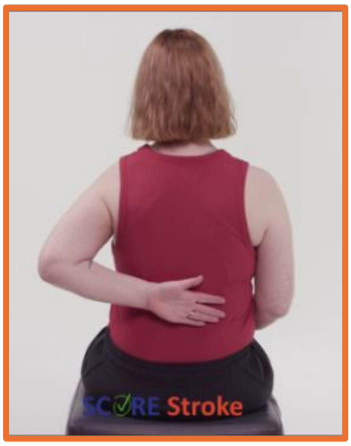

Start position

- ☑ Sitting on the edge of the chair to leave space for the arm to reach the back
- ☑ Hand resting in lap

End position

- ☑ The dorsal part of the hand touches the lumbar spine with MCP joints past the spinous processes

Verbal instructions with concurrent physical demonstration

"Move forward on the chair and then place your hand on the lower part of your back."

Assistance can be provided to assist the patient to move forward on the chair prior to commencing the movement

Points for consideration

- Do not allow the patient to use gravitational tricks, momentum or compensatory trunk movements
- Wrist and finger positions are not considered in scoring

Scoring **ITEM 12**

**0**	Hand does not pass the anterior superior iliac spine (ASIS) or arm movement is only performed using gravitational tricks or compensatory movements
**1**	Any movement past the ASIS without compensatory movements, but does not reach the end position
**2**	The dorsal part of the hand touches the lumbar spine with MCP joints past the spinous processes, without compensation of the trunk

SHOULDER FLEXION 0°-90°

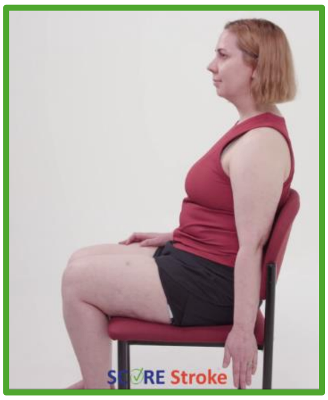

Start position

- ☑ Shoulder in neutral position at the side of the body
- ☑ Elbow fully extended to 0°
- ☑ Forearm in neutral mid-position of pronation-supination, palm facing the body
- ☑ Wrist in neutral position

End position

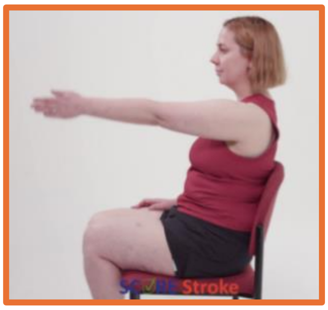

- ☑ Shoulder flexed to 90°
- ☑ Elbow extension maintained at 0°
- ☑ Forearm position maintained throughout the entire movement

Verbal instructions with concurrent physical demonstration

"Fully straighten your arm at the side of your body with your palm facing your hip and your elbow fully straightened. Now raise your arm to shoulder height keeping your elbow straight."

Assistance: No support is allowed to obtain or maintain the shoulder and elbow positions

Points for consideration

- Only unilateral performance is scored; however, the movement can be performed bilaterally to minimize compensations and for comparison with the non-affected arm
- Assess passive range of motion before scoring (use slow stretch if spasticity is present)
- An elbow extension deficit of up to 30° due to joint contracture (not spasticity) can be considered as patient's maximum available range of motion
- If elbow joint contracture is ≥30°, the item cannot be assessed correctly; mark the item 'not testable' and score 0
- Wrist and finger positions are not considered in scoring
- To score 1, full active elbow extension of available passive range of motion needs to be achieved and maintained when shoulder flexion begins

Scoring — ITEM 13

**0**	No shoulder flexion **OR**<br>cannot actively obtain and maintain the start position with a fully extended elbow when shoulder flexion begins (elbow flexion and/or shoulder abduction occurs immediately)
**1**	Performs only partially **OR**<br>maintains elbow extension at the beginning of the movement, but elbow flexion and/or shoulder abduction occurs before reaching 90° of shoulder flexion
**2**	Pure shoulder flexion to 90° without abduction while maintaining full elbow extension

PRONATION-SUPINATION, ELBOW 90° FLEXION

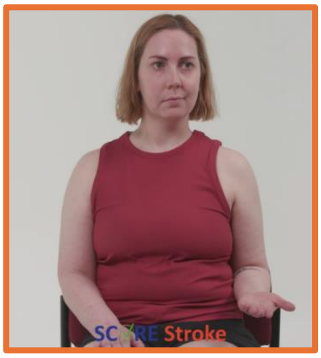

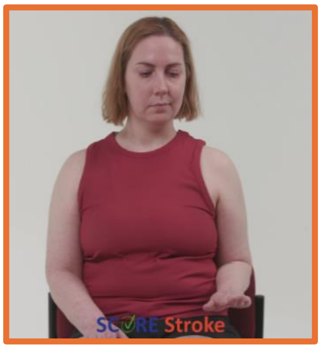

Start position

- ☑ Shoulder in neutral position (approximately 0° in all degrees of freedom)
- ☑ Elbow flexed to approximately 90°
- ☑ Forearm in neutral position between pronation and supination
- ☑ Wrist in neutral position

End position

- ☑ Shoulder position maintained during the movement
- ☑ Elbow flexion maintained at approximately 90°
- ☑ Full supination and pronation while the other joints of the arm are maintained stable

Verbal instructions with concurrent physical demonstration

"Bend your elbow with your hand in front of you. Now turn your palm up and down as far as you can while keeping your arm and the rest of your body still."

Assistance: No support is allowed to obtain or maintain the shoulder and elbow positions.

Points for consideration

- The initial shoulder and elbow positions need to remain stable during the pronation/supination movement
- Do not allow shoulder compensation (abduction or rotation) during performance
- Only unilateral performance is scored; however, the movement can be performed bilaterally to minimize compensations and for comparison with the non-affected arm
- Finger position and speed of movements are not considered in scoring
- To score 1, movement in both pronation and supination directions are required even with very limited range
- To score 2, full active pronation/supination of available passive range of motion is required

Scoring — ITEM 14

**0**	No pronation or supination occurs **OR**<br>cannot obtain and maintain the elbow and shoulder start position during movement even with very limited range **OR**<br>active movement occurs only in either pronation or supination
**1**	Performs only partially **OR**<br>does not reach full pronation and supination but maintains stable elbow and shoulder position **OR**<br>maintains start position in the beginning of the movement, but unable to maintain before reaching full pronation or supination
**2**	Full pronation and supination with maintained elbow and shoulder positions

A. IV. Movements with little or no synergy dependence

SHOULDER ABDUCTION 0°-90°

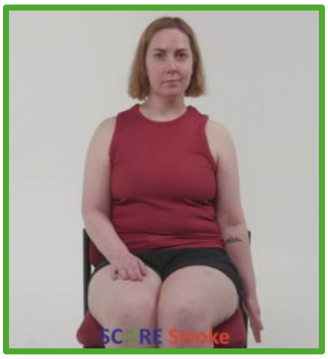

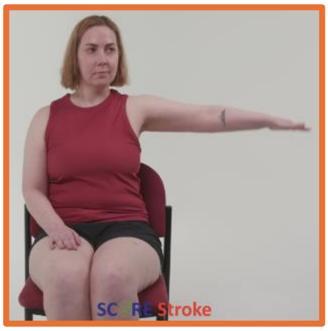

Start position

- ☑ Shoulder in neutral position at the side of the body
- ☑ Elbow fully extended to 0°
- ☑ Forearm in neutral mid-position of pronation-supination, palm faces the body
- ☑ Wrist in neutral position

End position

- ☑ Shoulder abducted to 90°
- ☑ Elbow extension maintained at 0°
- ☑ Forearm position maintained through the entire movement, palm faces the floor

Verbal instructions with concurrent physical demonstration

"Fully straighten your arm at the side of your body with your palm facing your hip and your elbow fully straightened. Now raise your arm to the side up to shoulder height, keeping your elbow straight."

Assistance: No support is allowed when the active shoulder abduction is initiated or performed

Points for consideration

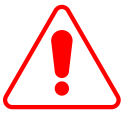

- Assess passive range of motion before scoring (use slow stretch if spasticity is present)
- An elbow extension deficit of up to 30° due to joint contracture (not spasticity) can be considered as patient's maximum available range of motion
- If elbow joint contracture is ≥30°, the item cannot be assessed correctly. Mark the item 'not testable' and score 0
- Wrist and finger positions are not considered in scoring
- Only unilateral performance is scored; however, the movement can be performed bilaterally to minimize compensations and for comparison with the non-affected arm
- Do not allow lateral compensations of the trunk or elevation of the shoulder girdle to reach shoulder abduction
- To score 1, full active elbow extension of available passive range of motion needs to be obtained and maintained when shoulder abduction begins

Scoring **ITEM 15**

**0**	No shoulder abduction **OR**<br>Cannot actively obtain and maintain the start position with fully extended elbow when shoulder flexion begins (elbow is flexed and/or forearm supinated immediately)
**1**	Performs only partially **OR**<br>Maintains elbow extension in the beginning of the movement, but elbow flexion and/or forearm supination occurs before reaching 90° of shoulder flexion
**2**	Pure shoulder abduction to 90° while maintaining elbow extension and forearm position during the movement

SHOULDER FLEXION 90°-180°

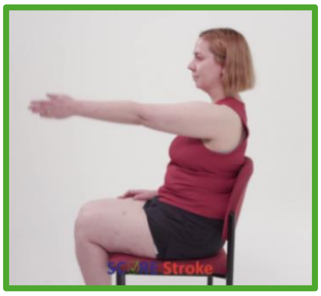

Start position

- ☑ Shoulder flexed to 90°
- ☑ Elbow fully extended to 0°
- ☑ Forearm in neutral mid-position between pronation-supination
- ☑ Wrist in neutral position

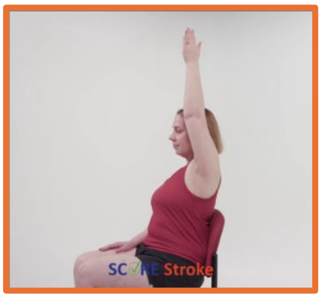

End position

- ☑ Shoulder fully flexed to maximum range of motion
- ☑ Elbow extension maintained at 0°
- ☑ Forearm position maintained through the entire movement

Verbal instructions with concurrent physical demonstration

"Sit up straight and lift your arm in front of your body to your shoulder height with your elbow fully straightened and your thumb facing upward. Now raise your arm up over your head as far as you can while keeping your elbow straight.

Assistance: No support is allowed when active shoulder flexion is initiated or performed

Points for consideration

- Assess elbow and shoulder passive range of motion before scoring (use slow stretch if spasticity is present)
- An elbow extension deficit of up to 30° due to joint contracture (not spasticity) can be considered as patient's maximum available range of motion
- If elbow joint contracture is ≥30°, the item cannot be assessed correctly; mark the item 'not testable' and score0
- Shoulder flexion active range of motion is about 160° across various age groups in community living non-disabled groups[1]
- Only unilateral performance is scored; however, the movement can be performed bilaterally to minimize compensations and for comparison with the non-affected arm
- Wrist and finger positions are not considered in scoring
- To score 1, full active elbow extension of available passive range of motion needs to be achieved and maintained when shoulder flexion begins

Scoring **ITEM 16**

**0**	No shoulder flexion beyond 90° **OR**<br>Cannot actively obtain and maintain the start position with elbow fully extended when shoulder flexion begins (elbow is flexed and/or shoulder abducted immediately when shoulder flexion begins)
**1**	Performs only partially **OR**<br>Maintains elbow extension in the beginning of the movement, but elbow flexion and/or shoulder abduction occurs before reaching full shoulder flexion
**2**	Full pure shoulder flexion while maintaining full elbow extension and without shoulder abduction during the movement

[1] Gill, T.K., Shanahan, E.M., Tucker, G.R. *et al.* Shoulder range of movement in the general population: age and gender stratified normative data using a community-based cohort. *BMC Musculoskelet Disord* **21**, 676 (2020). https://doi.org/10.1186/s12891-020-03665-9

PRONATION-SUPINATION, ELBOW 0° EXTENSION

Start position

- ☑ Shoulder flexed to approximately 30°
- ☑ Elbow extended to 0°
- ☑ Forearm in neutral mid-position between pronation and supination
- ☑ Wrist in neutral position

Full pronation

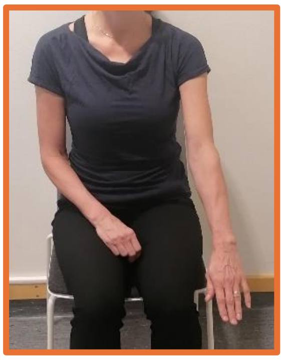

End position

- ☑ Shoulder flexion maintained at approximately 30°
- ☑ Elbow extension maintained at 0°
- ☑ Wrist in neutral position
- ☑ Full supination and pronation while the other joints of the arm are stable

Full supination

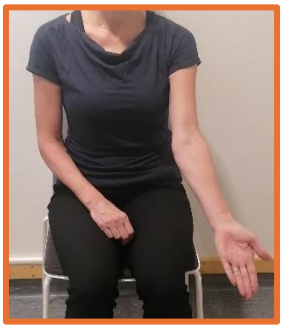

Verbal instructions with concurrent physical demonstration

"Lift your arm in front of you with your elbow straight. Now turn your palm up and down as far as you can while keeping your elbow straight."

Assistance: No support is allowed to take or maintain the shoulder and elbow positions during supination/pronation

Points for consideration

- Shoulder and elbow positions need to remain stable during the pronation/supination movement
- In full elbow extension, a cubitus angle (elbow carrying angle) between 5-15 degrees is normal
- An elbow extension deficit of up to 30° due to joint contracture (not spasticity) can be considered as patient's maximum available range of motion
- If elbow joint contracture is ≥30°, the item cannot be assessed correctly; mark the item 'not testable' and score 0
- Only unilateral performance is scored; however, the movement can be performed bilaterally to minimize compensations and for comparison with the non-affected arm
- Do not allow compensatory movements of the trunk or shoulder during performance
- Finger positions or speed of movements are not considered in scoring
- To score 1, pronation and supination movements are both required even with very limited range
- To score 2, full pronation/supination within available passive range of motion is required

Scoring — ITEM 17

**0**	No pronation and/or supination occurs **OR**<br>Cannot actively obtain and maintain the start position with fully extended elbow when the supination or pronation begins **OR**<br>Active movement occurs only in either pronation or supination
**1**	Performs only partially **OR**<br>Does not reach full pronation and supination but maintains elbow extension and stable shoulder position **OR**<br>Maintains start position in the beginning of the movement, but unable to maintain before reaching full pronation or supination
**2**	Full pronation and supination with maintained elbow and shoulder positions

A. V. Normal reflex activity

- Assessed only if full score of 6 points was achieved in the previous section A. IV. Movements with little or no synergy dependence
- If score < 6 in section A. IV, the score in this section is marked as 0
- The reflexes are scored in the same way as described in A. I. Reflex activity (Page 6)
- Reflex activity is tested in flexors (Biceps and finger flexors) and extensors (Triceps)

Points for consideration

- Test reflex on the non-affected side first; to use as a comparison
- The reflexes can be verified either visually or by palpation of the tendon
- A markedly hyperactive reflex is when the response is very easily or almost spontaneously elicited OR when the response is markedly stronger compared to the non-affected side
- Lively reflex response can be considered as a lower grade response compared to 'markedly hyperactive' or compared to the non-affected side

Scoring **ITEM 18**

**0**	At least 2 of the 3 reflexes are markedly hyperactive **OR**<br>scored less than 6 points in part IV
**1**	1 reflex markedly hyperactive OR at least 2 reflexes lively
**2**	Maximum of 1 reflex lively and none markedly hyperactive

B. Wrist

WRIST STABILITY AT 15° DORSAL EXTENSION, ELBOW 90°

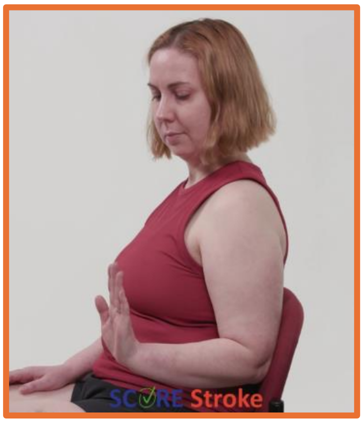

Start position

- ☑ Shoulder in neutral position (approximately 0° in all degrees of freedom)
- ☑ Elbow flexed to approximately 90°
- ☑ Forearm pronated
- ☑ Wrist in resting position
- ☑ Fingers can be extended, flexed or relaxed

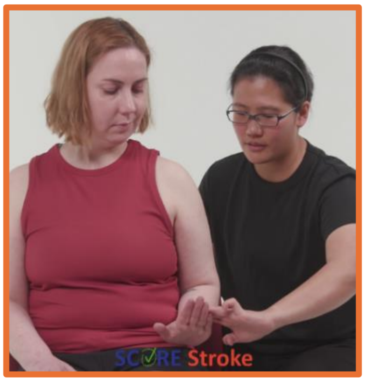

End position

- ☑ Shoulder in neutral position
- ☑ Elbow flexed to approximately 90°
- ☑ Forearm pronated
- ☑ Dorsal extension of the wrist to at least 15°

Verbal instructions with concurrent physical demonstration

"Bend your elbow with your hand in front of you. Now bend your wrist and hand upward. Keep your hand in this position while I apply slight pressure."

Assistance can be provided to maintain the required elbow and shoulder positions. No support is allowed close to the wrist or hand.

Resistance: When 15° of wrist extension is achieved, the assessor applies submaximal force (e.g. by using index and middle finger) to the dorsal hand, across the metacarpals, against wrist extension. The assessor stabilizes the forearm with their other hand.

Points for consideration

- Finger positions are not considered in scoring
- Active wrist extension of 15° is assessed visually (no goniometer required)
- To score 2, the resistance tolerated can be compared to the non-affected arm

Scoring **ITEM 19**

**0**	Cannot perform wrist dorsal extension of at least 15°
**1**	Achieves 15° of wrist dorsal extension, but cannot maintain this position against resistance
**2**	Maintains 15° of dorsal extension when resistance is applied

REPEATED WRIST DORSAL EXTENSION AND VOLAR FLEXION, ELBOW 90°

Start position

- ☑ Shoulder in neutral position (approximately 0° in all degrees of freedom)
- ☑ Elbow flexed to approximately 90°
- ☑ Forearm pronated
- ☑ Wrist in resting position
- ☑ Fingers can be extended, flexed or relaxed

Full dorsal extension

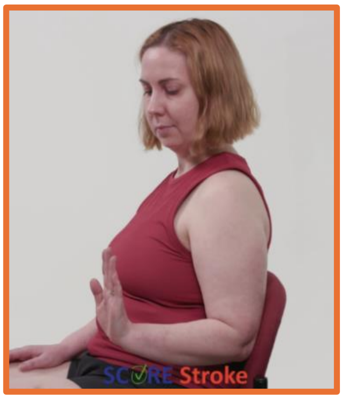

End position

- ☑ Shoulder in neutral position
- ☑ Elbow flexed to approximately 90°
- ☑ Forearm pronated
- ☑ Repeated full wrist dorsal extension and volar flexion

Full volar flexion

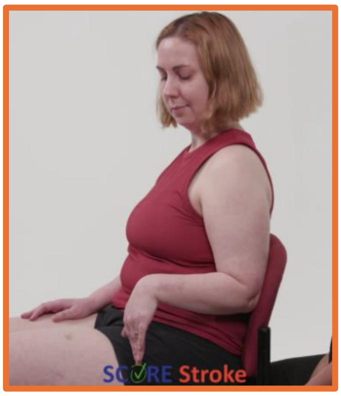

Verbal instructions with concurrent physical demonstration

"Bend your elbow with your hand in front of you. Now bend your wrist and hand up and down as far as you can several times."

Assistance can be provided to maintain the required elbow and shoulder positions. No support is allowed close to the wrist or hand.

Points for consideration

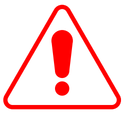

- Only unilateral performance is scored; however, the movement can be performed bilaterally to minimize compensations and for comparison with the non-affected arm
- Finger positions or speed of movements are not considered in scoring
- Do not allow compensatory movements of the elbow or shoulder during performance
- Fingers may be slightly flexed or extended during wrist movements
- To score 2, full active movement in both directions within passive range of motion is required
- To score 2, at least two complete alternated extension/flexion cycles are required

Scoring **ITEM 20**

**0**	No wrist dorsal extension and volar flexion **OR**<br>The wrist can only move in one direction
**1**	Performs only partially **OR**<br>Does not reach full passive range of motion but moves the wrist in both directions **OR**<br>Completes only one movement cycle
**2**	Completes at least two full range dorsal extension and volar flexion cycles in a repetitive manner

WRIST STABILITY AT 15° DORSAL EXTENSION, ELBOW 0°

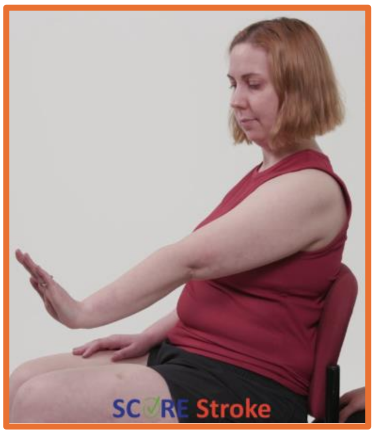

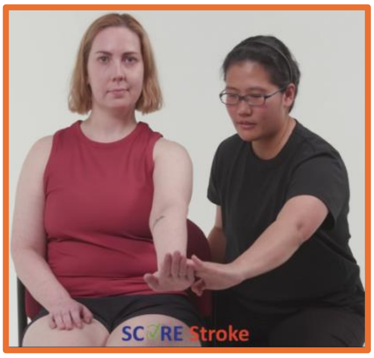

Start position

- ☑ Shoulder at approximately 30° flexion and abduction
- ☑ Elbow extended to 0°
- ☑ Forearm pronated
- ☑ Wrist in resting position
- ☑ Fingers can be extended, flexed or relaxed

End position

- ☑ Shoulder at approximately 30° flexion and abduction
- ☑ Elbow extended to 0°
- ☑ Forearm pronated
- ☑ Dorsal extension of the wrist to at least 15°

Verbal instructions with concurrent physical demonstration

"Lift your arm out in front of you with your elbow straight. Now bend your wrist and hand upward. Keep your hand in this position while I apply slight pressure."

Assistance can be provided to maintain the required elbow and shoulder positions. No support is allowed close to the wrist or hand.

Resistance: When 15° wrist extension is achieved the assessor can apply force by using two fingers (index and middle finger) to the dorsal hand, across metacarpals, against wrist extension.

Points for consideration

- Finger positions are not considered in scoring
- Active wrist extension of 15° is assessed visually (no goniometer required)
- If elbow joint contracture is ≥30°, the item cannot be assessed correctly; mark the item 'not testable' and score 0
- To score 2, resistance tolerated must be greater than or equal to the non-affected arm

Scoring

ITEM 21

**0**	Cannot perform wrist dorsal extension to at least 15°
**1**	Achieves 15° of wrist dorsal extension, but cannot maintain this position against resistance
**2**	Maintains 15° of dorsal extension when resistance is applied

REPEATED WRIST DORSAL EXTENSION AND VOLAR FLEXION, ELBOW 0°

Start position

- ☑ Shoulder at approximately 30° of flexion and abduction
- ☑ Elbow extended to 0°
- ☑ Forearm pronated
- ☑ Wrist in resting position
- ☑ Fingers can be extended, flexed or relaxed

Full dorsal extension

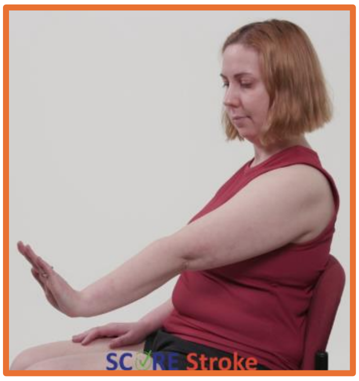

End position

- ☑ Shoulder at approximately 30° of flexion and abduction
- ☑ Elbow extended to 0°
- ☑ Forearm pronated
- ☑ Repeated full wrist dorsal extension and volar flexion

Full volar flexion

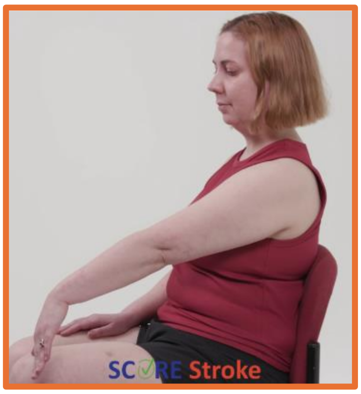

Verbal instructions with concurrent physical demonstration

“Lift your arm in front of you with your elbow straight. Now bend your wrist up and down as far as you can several times.”

Assistance can be provided to maintain the required elbow and shoulder positions. No support is allowed close to the wrist or hand.

Points for consideration

- Only unilateral performance is scored; however, the movement can be performed bilaterally to minimize compensations and for comparison with the non-affected arm
- If elbow joint contracture is ≥30°, the item cannot be assessed correctly; mark the item ‘not testable’ and score 0
- Do not allow compensatory movements of the elbow or shoulder during performance.
- Finger positions or speed of movements are not considered in scoring
- Fingers may be slightly flexed or extended during wrist movements
- To score 2, full active movement in both directions within passive range of motion is required
- To score 2 at least two complete alternated extension/flexion cycles are required

Scoring **ITEM 22**

**0**	No wrist dorsal extension and volar flexion **OR**<br>The wrist can only move in one direction
**1**	Performs only partially **OR**<br>Does not reach the full passive range of motion but moves the wrist in both directions **OR**<br>Completes only one movement cycle
**2**	Completes at least two full range dorsal extension and volar flexion cycles in a repetitive manner

CIRCUMDUCTION, ELBOW 90°

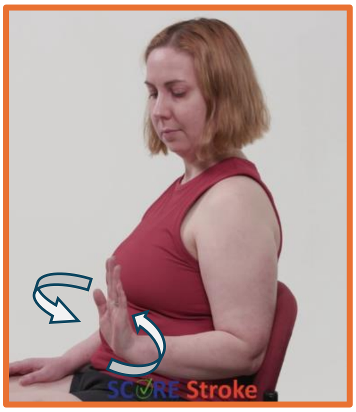

Start position

- ☑ Shoulder in neutral position (approximately 0° degrees in all degrees of freedom)
- ☑ Elbow flexed to approximately 90°
- ☑ Forearm pronated
- ☑ Wrist in neutral position
- ☑ Fingers can be extended, flexed or relaxed

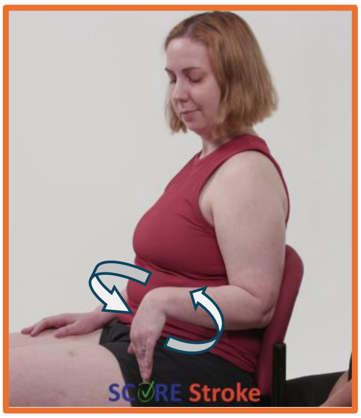

End position

- ☑ Shoulder in neutral position
- ☑ Elbow flexed to approximately 90°
- ☑ Forearm maintained in pronated position
- ☑ Completes full wrist circumduction without an extensive simultaneous supination or pronation of the forearm

Verbal instructions with concurrent physical demonstration

"Bend your elbow with your hand in front of you. Now make a full and smooth circle with your hand by moving the wrist, try to make as big a circle as possible."

Assistance can be provided to maintain the required elbow and shoulder positions. No support is allowed close to the wrist or hand.

Points for consideration

- Only unilateral performance is scored; however, the movement can be performed bilaterally to minimize compensations and for comparison with the non-affected arm
- Circumduction of the wrist needs to be a circular motion combining flexion, extension, and radioulnar deviation without excessive simultaneous supination or pronation of the forearm
- Circumduction can be performed in any direction
- Do not allow compensatory movements of the elbow or shoulder during performance.
- Finger positions or speed of movements are not considered in scoring
- To score 2, full circumduction within passive range of motion is required

Scoring **ITEM 23**

**0**	No circumduction possible
**1**	Performs only partially **OR**<br>Wrist circumduction incomplete or jerky (not smooth)
**2**	Full and smooth wrist circumduction

C. Hand

FINGER MASS FLEXION

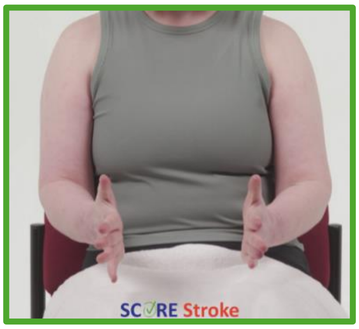

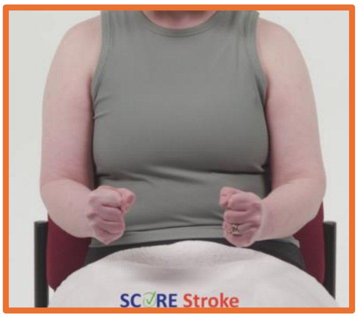

Start position

- ☑ Shoulder in neutral position
- ☑ Elbow flexed to approximately 90°
- ☑ Forearm and wrist in resting position
- ☑ Fingers fully extended (actively or passively)

End position

- ☑ Full finger flexion
- ☑ Thumb flexed and positioned outside the fist

Verbal instructions with concurrent physical demonstration

"Bend your elbow with your hand in front of you. Now open your hand, and then bend your fingers to make a full fist (thumb outside the fingers)."

Assistance can be provided to maintain the required elbow and shoulder positions. No support is allowed to the wrist or hand.

The assessor can passively extend the fingers before scoring to allow testing of active flexion from the fully extended position.

Points for consideration

- Finger flexion needs to be active; it cannot be achieved by a passive recoiling of fingers e.g., through finger flexor spasticity or a passive tenodesis grip
- The anatomically combined wrist dorsal extension is permitted during the active finger flexion

Scoring **ITEM 24**

**0**	No flexion in any fingers
**1**	Performs only partially, any degree of finger flexion that is less than needed to make a full fist **OR**<br>Not all fingers can be flexed OR thumb inside the fist hindering full flexion of fingers 2-5
**2**	Full flexion of all 5 fingers with the fingertips touching the palm, thumb outside the fist

FINGER MASS EXTENSION

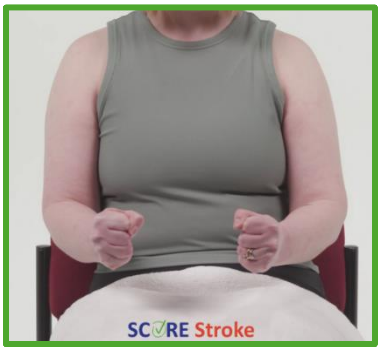

Start position

- ☑ Shoulder in neutral position
- ☑ Elbow flexed to approximately 90°
- ☑ Forearm and wrist in resting position
- ☑ Fingers fully flexed (actively or passively)

End position

- ☑ All 5 fingers are extended fully

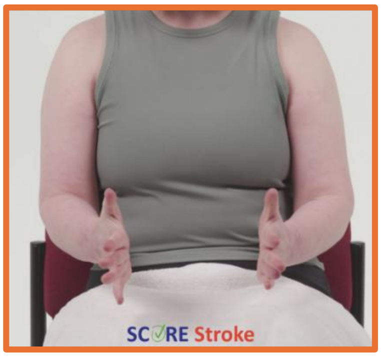

Verbal instructions with concurrent physical demonstration

"Bend your elbow with your hand in front of you and form a full fist. Now open your hand and straighten all your fingers as much as possible."

Assistance can be provided to maintain the required elbow and shoulder positions. No support is allowed to the wrist or hand.

The assessor can passively flex the fingers before scoring to allow testing of active extension from the fully flexed position.

Points for consideration

- Finger extension needs to be active, e.g. it cannot be achieved by a passive tenodesis grip
- The anatomically combined wrist volar flexion is permitted during the active finger extension

Scoring

ITEM 25

**0**	No finger extension in any fingers
**1**	Performs only partially, any degree of finger extension that is less than full extension **OR**<br>Not all fingers can be extended
**2**	Full extension of all 5 fingers

HOOK GRASP

Start position

- ☑ Shoulder in neutral position
- ☑ Elbow flexed to approximately 90°
- ☑ Forearm, wrist and fingers in resting position

View from above

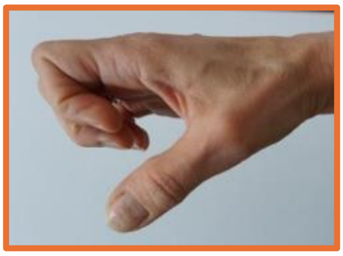

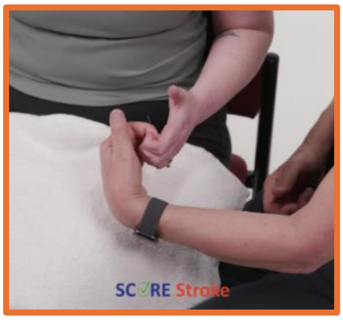

End position

- ☑ The MCP joints in digits 2-5 extended to 0°
- ☑ The proximal interphalangeal (PIP) joints and distal interphalangeal (DIP) joints in digits 2-5 flexed to form a hook position

Verbal instructions with concurrent physical demonstration

"Straighten the knuckles of your hand fully while bending the fingers, make your hand look like a hook or claw. Hold this position while I try to draw your fingers open."

Assistance can be provided to maintain the required elbow and shoulder positions. No support is allowed to the wrist or hand.

Resistance is applied against the fingertips and tested only after the correct hook position has been achieved

Points for consideration

- The hook position needs to be obtained actively and independently by the patient
- Thumb, wrist and forearm positions are not considered in scoring
- To score 1 or 2, MCP joints need to remain extended while the PIP and DIP joints are flexed

Scoring **ITEM 26**

**0**	Cannot actively achieve the required hook hand position
**1**	Can achieve the required hook hand position, but can't maintain the hook position against resistance
**2**	Holds the hook grasp against moderate resistance applied by the assessor (pulling away from the hand)

THUMB ADDUCTION

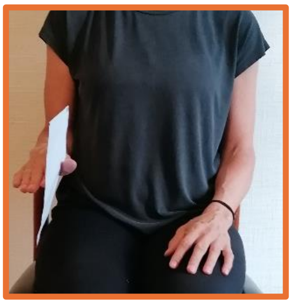

Start position

- ☑ Shoulder in neutral position
- ☑ Elbow flexed to approximately 90°
- ☑ Forearm pronated, wrist and fingers in resting position

End position

- ☑ Pure thumb adduction with carpometacarpal (CMC) and interphalangeal joints of the thumb, MCP, PIP and DIP joints extended at 0°
- ☑ Holds paper between the thumb and MCP joint of the index finger, flat hand with the palm facing downward

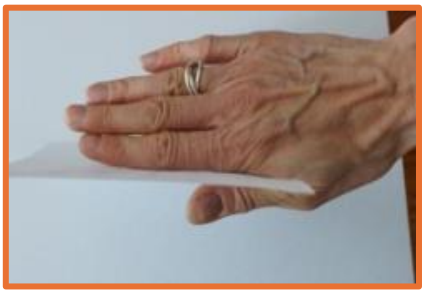

View from above

Verbal instructions with concurrent physical demonstration

"Hold your hand palm down and straighten your fingers. Bring your thumb to meet your other fingers. Now hold this paper between your thumb and index finger. Keep your fingers and knuckles straight while I gently try to pull the paper away."

Assistance can be provided to maintain the required elbow and shoulder positions. No support is allowed to the wrist or hand.

Only when an active thumb adduction is observed, an A5 size piece of paper is presented close to the hand so that the patient can actively grasp the paper between the index finger and thumb and hold it against gravity.

For score 2, the assessor pulls the paper away from the hand by applying a slight **horizontal** tug.

Points for consideration

- The thumb needs to be in the same plane as the rest of the hand (pure thumb adduction)
- The required hand position (thumb adducted and hand flat) needs to be maintained when the slight horizontal tug is applied
- Wrist and forearm positions are not considered in scoring
- For score 1 the paper needs to be held against gravity

Scoring **ITEM 27**

**0**	Cannot perform pure thumb adduction with fully extended thumb and finger joints
**1**	Can achieve a pure thumb adduction and the piece of paper between the extended thumb and lateral side of the 2nd metacarpal while maintaining fully extended thumb and finger joints
**2**	Maintains the pure thumb adduction with fully extended thumb and finger joints while holding the paper against a slight tug

PINCER GRASP, OPPOSITION

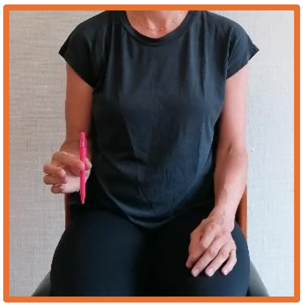

Start position

- Shoulder in neutral position
- Elbow flexed to approximately 90°
- Forearm, wrist and fingers in resting position
- Hand in front of the body ready to grasp the pen/pencil

End position

- The thumb and index finger are actively extended in preparation to grasp the pen/pencil presented close to the hand and fingers
- The pads (pulp) of the thumb and index finger are opposed and holding the pen/pencil

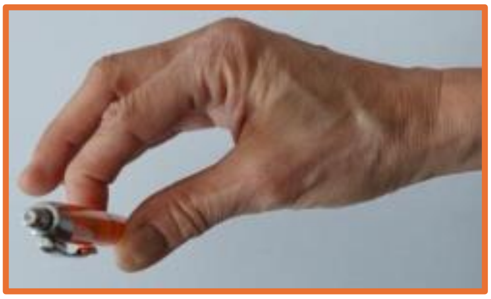

View from above

Verbal instructions with concurrent physical demonstration

"Take the pen/pencil from me and hold it between the pads of your thumb and index finger. Hold firmly while I pull it up."

Assistance can be provided to maintain the required elbow and shoulder positions. No support is allowed to the wrist or hand.

The assessor presents a pen/pencil in a vertical position close to the patient's hand so that the patient can actively grasp the pen/pencil and hold it between the index finger and the thumb.

For score 2, the assessor pulls the pen/pencil upward in a vertical direction by applying a submaximal force.

Points for consideration

- The grasp needs to be active
- Ensure that the pencil is in contact with the pads of the thumb and index finger and not against the lateral side of the index finger
- Digits 2-5 should not support the thumb and index fingers pincer grip
- The movement needs to be performed unilaterally, help from the patient's non-affected hand is not allowed
- Wrist and forearm positions are not considered in scoring
- Score 0 if the initial grasp cannot be maintained (for example the pen/pencil glides away from the fingertip pincer grip)

Scoring **ITEM 28**

**0**	Cannot actively open the fingers to grasp or hold the pencil between the pads of the thumb and index finger
**1**	Can grasp and hold the pen/pencil, but cannot hold against a tug
**2**	Can grasp and hold the pen/pencil against an upward tug

CYLINDER GRASP

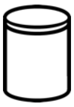

Start position

- ☑ Shoulder in neutral position
- ☑ Elbow flexed to approximately 90°
- ☑ Forearm, wrist and fingers in resting position
- ☑ Hand in front of the body ready to grasp the can

End position

- ☑ The fingers are actively extended in preparation to grasp the can presented close to the hand and fingers
- ☑ Holds the can with opposed thumb and all fingers around the can; the volar surface of the hand is in contact with the can to form a power cylinder grip

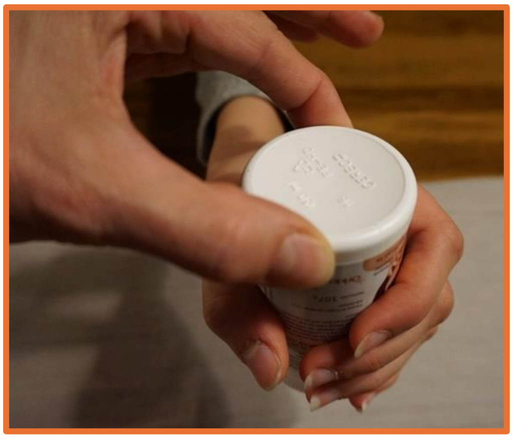

The assessor hand is holding the top of the can to apply a tug

Verbal instructions with concurrent physical demonstration

"Grasp and hold the can with your fingers and palm tightly around it and your thumb on the other side. Hold firmly while I pull it."

Assistance can be provided to maintain the required elbow and shoulder positions. No support is allowed to the wrist or hand.

The assessor presents a can close to the patient's hand so that the patient can actively grasp the can and hold it.

For score 2, the assessor pulls the can **upwards** by applying a submaximal force

Points for consideration

- The grasp needs to be active
- The movement needs to be performed unilaterally, help from the patient's non-affected hand is not allowed
- Wrist and forearm positions are not considered in scoring
- An empty hard plastic container (such as a spice container) approximately 5 cm in diameter can be used

Scoring **ITEM 29**

**0**	Cannot actively open the fingers to grasp or hold the can
**1**	Can grasp and hold the can with the palm of the hand in contact with the can and thumb opposed, but cannot hold against a tug
**2**	Can grasp and hold the can against an upward tug

SPHERICAL GRASP

Start position

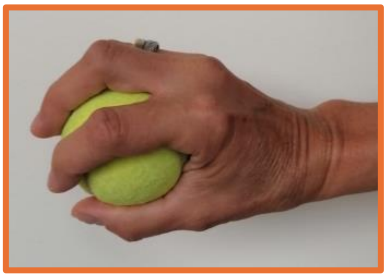

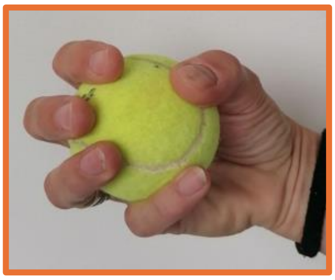

- Shoulder in neutral position
- Elbow flexed to approximately 90°
- Forearm, wrist and fingers in resting position
- Hand in front of the body ready to grasp the tennis ball

End position

- The fingers are actively extended in preparation to grasp the ball presented close to the hand and fingers
- Holds the ball with opposed thumb and fingers slightly abducted around the ball; the volar surface of the hand is in contact with the ball to form a power spherical grip

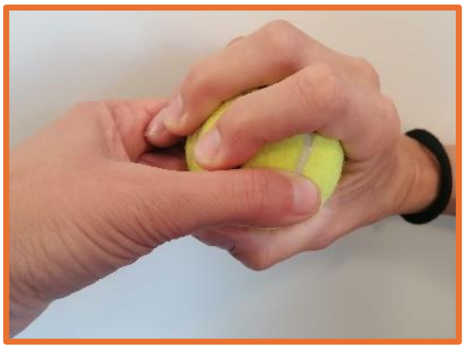

The assessor hand (on the left-hand side) is pulling the ball out of the patient's hand

Verbal instructions with concurrent physical demonstration

"Grasp the ball and hold it in your hand with your fingers and thumb opposite of each other; hold firmly while I pull it."

Assistance can be provided to maintain the required elbow and shoulder positions. No support is allowed to the wrist or hand.

The assessor presents a tennis ball to the open palm close to the patient's hand so that the patient can actively grasp the ball and hold it against gravity.

For score 2, the assessor pulls the ball **out from the hand** towards the patient's fingertips by applying a submaximal force.

Points for consideration

- The grasp needs to be active
- To confirm sufficient thumb opposition, the ball needs to be visible in the space between the thumb and index finger
- The movement needs to be performed unilaterally, help from the patient's non-affected hand is not allowed
- Wrist and forearm positions are not considered in scoring

Scoring **ITEM 30**

**0**	Cannot actively open the fingers to grasp or hold the ball
**1**	Can grasp and hold the ball with the palm of the hand in contact with the ball and thumb opposed, but cannot hold against a tug
**2**	Can grasp and hold the ball against a pull

D. Coordination – speed

☑ The finger-to-nose test needs to be completed 5 times with eyes closed or blindfolded

☑ Confirm that the patient can reach and touch their nose with the tip of their index finger

☑ The finger-to-nose movement cycle needs to be completed 5 times, if not, all three items are scored 0

☑ A complete finger-to-nose movement: 1) starts with the hand resting on the knee, 2) the tested hand moves to touch the nose with the tip of the index finger, 3) the movement ends when the hand is back in the starting position on the knee

☑ Use practice trials to ensure that the patient understood the instructions

☑ If needed the assessor can count the 5 repetitions out loud

☑ Begin timing when the hand leaves the knee and stop when the hand touches the knee after 5 repetitions

☑ Record the time for both arms by timing the non-affected arm first; the difference in time between the arms is used in scoring

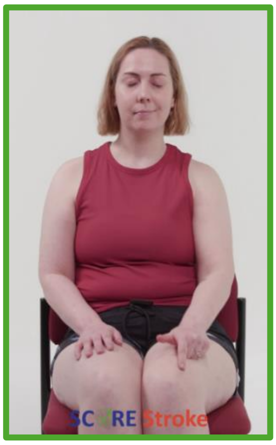

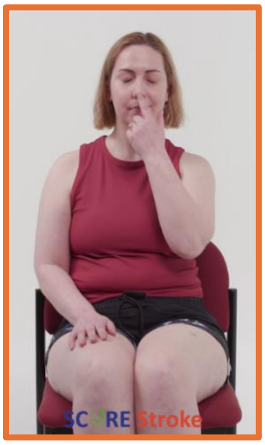

Start and end position

☑ Sitting upright

☑ Hand is resting on the ipsilateral knee

☑ Eyes closed or blindfolded

Finger position on the nose

☑ The tip of the index finger touching the tip of the nose

Verbal instructions with concurrent physical demonstration

"Sit up straight with your hand on your knee and keep your eyes closed. Bring the tip of your index finger to the tip of your nose 5 times, as fast and precisely as possible. I am going to time you."

Assistance: Not allowed

Points for consideration

- Do not allow movement of the head or trunk to compensate for limited arm movement
- Other fingers can be flexed or extended as long as the index finger touches the nose
- **Tremor** is assessed as any deviation from a straight and smooth movement trajectory [2]
- **Dysmetria** is assessed as any deviation from the tip of the index finger touching the tip of the nose
- **Slight dysmetria** is scored when the fingertip touches a close circle around the nose
- **Pronounced dysmetria** is scored when the finger touch is further away from the nose, e.g., on the cheek, given that the patient can touch the nose when movement is performed slowly
- Score 2 if only first movement shows slight deviation of the nose; and all following 4 movements point correctly on the nose.
- Dysmetria is not scored for the touch on the knee, but the hand needs to touch the knee close to the starting position when returning from the nose
- Score 0 if the dysmetria is either pronounced or unsystematic

[2] Rodrigues MR, Slimovitch M, Chilingaryan G, Levin MF. Does the Finger-to-Nose Test measure upper limb coordination in chronic stroke? J Neuroeng Rehabil. 2017 Jan 23;14(1):6.

Scoring

Tremor **ITEM 31**

**0**	Marked tremor that substantially interferes with coordination **OR**<br>Unable to complete 5 repetitions of the task
**1**	Slight tremor, that mildly interferes with coordination
**2**	No tremor is observed during all 5 repetitions

Dysmetria **ITEM 32**

**0**	Pronounced or unsystematic dysmetria (points on the cheek or points randomly on different locations) **OR**<br>Unable to complete 5 repetitions of the task
**1**	Slight but systematic dysmetria (points systematically in the same location close to the tip of the nose)
**2**	No dysmetria (the tip of the index finger lands on the tip of the nose all 5 repetitions)

Time **ITEM 33**

**0**	The affected arm is 6 or more seconds slower than the non-affected arm **OR**<br>Unable to complete 5 repetitions of the task
**1**	The affected arm is 2.0 to 5.9 seconds slower than the non-affected arm
**2**	The affected arm is less than 2 seconds slower (or faster) than the non-affected arm

Appendix. Quick guide on assistance and support allowed

FMA-UE items	Assistance during active movement	Details on assistance and support
**A. SHOULDER / ELBOW / FOREARM**		
**II. MOVEMENTS WITHIN SYNERGIES**		
Flexor Synergy	**NO**	Only allowed to move the arm into the start position.
Extensor Synergy	**NO**	Only allowed to move the arm into the start position. During the active synergy movement antigravitational resistance can be applied to ensure that the movement is active.
**III. MOVEMENTS WITH MIXED SYNERGIES**		
Hand to lumbar spine	**NO**	Only allowed to move the arm into the start position.
Shoulder flexion 0-90°	**NO**	Full passive range of elbow extension is assessed before scoring.
Pronation-Supination, elbow 90°	**NO**	Full passive range of elbow extension is assessed before scoring.
**IV. MOVEMENTS WITH LITTLE OR NO SYNERGY DEPENDENCE**		
Shoulder abduction 0-90°	**NO**	Full passive range of elbow extension is assessed before scoring.
Shoulder flexion 90°-180°	**NO**	Full passive range of elbow extension is assessed before scoring.
Pronation-supination, elbow 0°	**NO**	Full passive range of elbow extension is assessed before scoring.
**B. WRIST**		
Stability at 15° extension, elbow 90°	**Wrist/hand NO**<br>**Shoulder/elbow YES**	Support can be provided to assist the patient to maintain the elbow and shoulder positions during the wrist movements. No assistance/support at the wrist or hand is allowed.<br>For stability items slight resistance with 2 fingers is applied on the back of the hand, close to the MCP joints. For maximum score of 2, the resistance of assessor's force applied by 2 fingers needs to be tolerated by the patient. A stabilizing force can be applied on the forearm when testing stability.
Repeated extension/flexion, elbow 90°		
Stability at 15° extension, elbow 0°		
Repeated extension/flexion, elbow 0°		
Circumduction		
**C. HAND and GRASP**		
Mass flexion	**Wrist/hand: NO**<br>**Shoulder/elbow: YES**	Support can be provided to assist the patient to maintain the elbow and shoulder positions. No assistance/support at the wrist or hand is allowed.<br>**Mass flexion/extension:** The assessor can passively extend or flex the fingers, respectively, before testing active movement.<br>**GRASP:** For score 2, the applied force can be compared with the non-affected side. The force applied for hook, pen, can and ball grasps is submaximal and slight for paper grasp. The direction of the force is out from the hand (paper away from the hand towards the assessor, pencil and can upward, ball away from the fingers).
Mass extension		
Hook grasp		
Thumb adduction		
Pincer grasp		
Cylinder grasp		
Spherical grasp		
**D. COORDINATION / SPEED**		
Coordination/speed	**NO**	Only allowed to move the arm into the start position.
